# Myocardial Afterload Is a Key Biomechanical Regulator of Atrioventricular Myocyte Differentiation in Zebrafish

**DOI:** 10.3390/jcdd9010022

**Published:** 2022-01-12

**Authors:** Neha Ahuja, Paige Ostwald, Alex Gendernalik, Elena Guzzolino, Letizia Pitto, David Bark, Deborah M. Garrity

**Affiliations:** 1 Program in Cell and Molecular Biology and Department of Biology, Colorado State University, Fort Collins, CO 80523, USA; neha.ahuja@utsouthwestern.edu (N.A.); postwald@rams.colostate.edu (P.O.); 2 School of Biomedical Engineering, Colorado State University, Fort Collins, CO 80523, USA; Alex.Gendernalik@colostate.edu; 3 Department of Pediatric Cardiology, Meyer Children’s Hospital, 50139 Florence, Italy; elena.guzzolino@gmail.com; 4 Institute of Clinical Physiology, National Council of Research, 56100 Pisa, Italy; l.pitto@ifc.cnr.it; 5 Department of Pediatrics, Washington University School of Medicine, St. Louis, MO 63110, USA; David.Bark@colostate.edu

**Keywords:** developmental biology, hemodynamics, afterload, heart, biomechanics, valve, zebrafish

## Abstract

Heart valve development is governed by both genetic and biomechanical inputs. Prior work has demonstrated that oscillating shear stress associated with blood flow is required for normal atrioventricular (AV) valve development. Cardiac afterload is defined as the pressure the ventricle must overcome in order to pump blood throughout the circulatory system. In human patients, conditions of high afterload can cause valve pathology. Whether high afterload adversely affects embryonic valve development remains poorly understood. Here we describe a zebrafish model exhibiting increased myocardial afterload, caused by vasopressin, a vasoconstrictive drug. We show that the application of vasopressin reliably produces an increase in afterload without directly acting on cardiac tissue in zebrafish embryos. We have found that increased afterload alters the rate of growth of the cardiac chambers and causes remodeling of cardiomyocytes. Consistent with pathology seen in patients with clinically high afterload, we see defects in both the form and the function of the valve leaflets. Our results suggest that valve defects are due to changes in atrioventricular myocyte signaling, rather than pressure directly acting on the endothelial valve leaflet cells. Cardiac afterload should therefore be considered a biomechanical factor that particularly impacts embryonic valve development.

## 1. Introduction

Congenital heart defects (CHD) occur when the heart fails to form appropriately during the early stages of embryonic development. Approximately 1.35 million newborns born annually with CHD worldwide [1]. Valve pathology occurs in over 50% of all CHD cases [2]. Despite the prevalence of CHD, the etiology of CHD is not well understood. In fact, only approximately 20% of all CHD arise from a known genetic lesion [3]. Phenotypically-similar CHD may arise from mutations in unrelated proteins [4], and conversely, identical mutations can cause a variety of distinct phenotypes [5]. A likely explanation of this phenomenon is that the pathology arises not only from loss or modification of protein function, but also through the alteration of the mechanical environment of the heart. Increasingly, research efforts are directed at defining biomechanical contributions to normal heart development and CHD. 

Shear stress produced by blood flow within the heart lumen, contraction itself, and pressures resulting from contraction, represent types of biomechanical cues that facilitate the normal development of the valves [6,7]. Because endothelial cells contributing to development of the AV valve are in direct contact with blood flow, they may be especially sensitive to perturbations in hemodynamics. As one example, exposure of porcine valves to physiological levels of shear stress was found to directly impact the development of the striated ECM present in the valve leaflets through the induction of collagen expression and even differentially affects each forming valve leaflet [8]. 

While the biomechanical signals derived from shear stress are increasingly well characterized, the impact of other potential biomechanical signals, such as cardiac loading on cardiac development, remain relatively uninvestigated. Afterload refers to the resistance the ventricle must overcome in order to pump blood throughout the circulatory system. For a given cardiac output, the pressure is proportional to the flow rate times the resistance. The higher the afterload, the higher the transmural pressure throughout the heart [9]. 

In the fetus, conditions of high afterload have been linked to ventricular hypertrophy and fetal hydrops [10]. Human fetuses with decreased fetal growth demonstrate decreased cardiac output thought to be caused by increased afterload [11], as well as tricuspid valve regurgitation [10]. Moreover, afterload is linked to CHD as shown by work demonstrating that reduction in afterload through hydralazine (a vasodilator) treatment can help to mitigate large ventricular septal defects [12,13]. The impact of high afterload has been best characterized in adult organisms, where its deleterious effects lead to valve pathology and cardiac hypertrophy [14,15,16]. Specifically, adult patients with conditions of increased afterload often experience concentric pathologic hypertrophy, in which sarcomeres are added in parallel within existing myofibrils [17]. A better understanding of the molecular circuitry activated by the afterload-response may yield translational insights into the causes of both adult valve pathology and CHD. 

Studies of how abnormal afterload might impact embryonic valve development so far have been hampered by a paucity of in vivo tools able to accurately and reliably induce the conditions of altered loading without directly impairing cardiac contraction. Prior work has utilized models such as outflow tract (OFT) ligation in chick embryos to increase afterload [18]. Increasing afterload through OFT ligation has resulted in a smaller mitral valve, dysregulation of shear-stress-responsive genes and altered expression of ECM markers [19]. Conversely, computational models of avian embryonic valve development have predicted that increased pressure on the developing endocardial cushions would promote leaflet-like elongation. These studies demonstrate that loading can affect AV valve development.

In order to respond to changes in afterload, cells at the atrio-ventricular junction (AVJ) must be able to perceive changes in blood flow and pressure. Endocardial cells can sense both the direction and the magnitude of blood flow through mechanosensors [7]. The monocilia present on endocardial cells are thought to bend under conditions of high flow and shear stress, leading to the activation of signaling pathways. Mouse endocardial cells lacking cardiac cilia fail to properly develop endocardial cushions and show impaired endothelial-to-mesenchymal transition (EMT) [20]. Mechanosensitive ion channels can activate expression of transcription factor *klf2a* under conditions of oscillatory flow [21]. Despite being physically separated from flow, myocardial cells also play a role in mechanotransduction. In vitro rat myocardial cells adapted to changes in load by undergoing hypertrophy [15]. Myocardial cells may perceive changes in load by assessing their degree of stretch using NFAT–calcineurin or titan-mediated mechanisms [22]. These cells may also sense the direct pressure transmitted through the cardiac wall through integrin-mediated mechanisms [22]. As part of their normal function, myocardial cells release paracrine signaling factors, such as BMP, that are required to signal endocardial differentiation [23]. Based on these findings, it is possible that the loading state of the myocytes impacts their release of key signals required for normal valve development.

In the circulatory system, resistance is mathematically described as Resistance=(8μL)/(π*r*^4^); where µ is the fluid viscosity, *L* is the length of the vessel, and *r* is the radius of the vessel. Because of this relationship, very small changes in the radius of the vasculature can have a dramatic impact on the resistance (and afterload) the heart encounters. We therefore chose to use vasopressin, a potent vasoconstrictor, to increase afterload and evaluate the effects on AV valve development in embryonic zebrafish. 

Vasopressin is an amino-acid hormone that causes vasoconstriction. The zebrafish genome encodes the following three vasopressin receptors: *avtr_1a1_*, *avtr_1a2_*, and *avpr2*. *Avtr_1a1_* and *avtr_1a2_* are both homologues of the mammalian V1a receptor [24]. The mammalian V1 receptor is expressed predominantly in the smooth muscle cells surrounding peripheral vessels. Vasopressin binding triggers an intracellular cascade that increases the intracellular calcium concentration and consequently causes muscle constriction and vasoconstriction [25]. The mammalian V2 receptor, which is a homolog of the zebrafish *avpr2* receptor, is expressed predominantly in the kidneys and acts to increase blood volume. Stimulation via either receptor raises blood pressure and directly increases myocardial afterload through increased vascular resistance [26]. 

Here, we show that vasopressin is vasoactive in zebrafish, and demonstrate that vasopressin application produces alterations in cardiac function and growth. We find that increased afterload affects the differentiation of both endocardial and myocardial valve cells. Our data support myocardial AVJ cells as especially sensitive to alterations in afterload in normal development.

## 2. Materials and Methods

### 2.1. Zebrafish Husbandry and Vasopressin Application

Zebrafish were raised as per Colorado State University animal care and use protocols. Zebrafish embryos were raised at 28.5 °C until 24 hpf in E3 medium, and dechorionated. Embryos were placed in a solution of arginine vasopressin acetate salt (Sigma, catalog number V9879, St. Louis, MO, USA) diluted in E3 embryo media to a final concentration of 10 µmol/L, unless otherwise specified. Treated embryos remained in the vasopressin solution for the entirety of the study. 

### 2.2. Live Imaging

For vessel and whole-heart live imaging, samples were immobilized in low-melting agarose. Quantification of vessel diameter was performed in Image J [27]. Quantification of cardiac chamber volume and looping angle was performed as previously described [28,29]. 

For live imaging of the valves, confocal microscopy was used. Embryos were treated with MESAB until cardiac contraction stopped, and then mounted in low-melt agarose in a depression slide in a tilted ventral-up orientation, such that the heart was roughly parallel to the coverslip. 

### 2.3. Quantification of Cardiac Functional Parameters

Quantification of cardiac parameters (cardiac output, stroke volume, and heart rate) was performed as previously described [30]. Briefly, embryos were raised at 28.5 °C until the appropriate time point. Fish were mounted in low-melt agarose, with no MESAB. Videos were taken with a Photron Mini UX-100 camera at 1600 frames per second, across at least 3 cardiac cycles. Videos were analyzed as previously described using a line scan and spatiotemporal kymographs to quantify cell movement along the line scan [30].

### 2.4. Immunohistochemistry

Immunohistochemistry was performed on dissected zebrafish hearts as previously described [31]. Briefly, hearts were dissected in an L15-10% FBS solution and then placed on a polylysine-coated slide. Hearts were fixed for 40 min in 4% PFA and permeabilized with a 1% solution of Triton X. For ALCAM labelling, zn8 antibody (Developmental Studies Hybridoma Bank) was used at 1:10 dilution. Embryos were washed 3xwith PBST solution and then incubated with Alexa-546 anti-mouse IgG secondary antibody (Thermo Fischer Cat #A-11030, Eugene, OR, USA) at a 1:200 dilution. Hearts were mounted in 50% glycerol under coverslips. Images were taken on the Zeiss LSM800 and analyzed blind. 

### 2.5. Reverse Transcriptase PCR

RNA was extracted from either dissected hearts or entire embryos, using TRIzol (Invitrogen, Carlsbad, CA, USA) according to manufacturer’s protocol and diluted in nuclease-free water (Sigma, St. Louis, MO, USA). cDNA synthesis was performed using AMV reverse transcriptase (Fisher Scientific, Waltham, MA, USA) and Oligo(dT)_12–18_ primer (Invitrogen, Carlsbad, CA, USA). PCR products were separated via a 2% agarose gel. For vasopressin receptor expression analysis, gene expression was scored only as present or absent based on presence of a band. 

## 3. Results

### 3.1. Vasopressin Actively Elicits Vasoconstriction in Zebrafish Embryos

**Figure 1:**
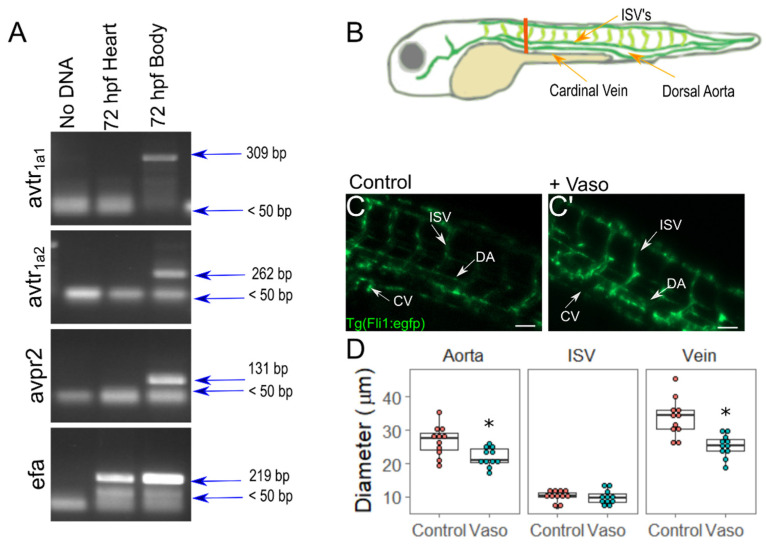
Application of vasopressin causes vasoconstriction of major blood vessels in zebrafish by 48 hpf. (**A**) RT-PCR of vasopressin receptors in whole body vs. cardiac tissues. (**B**) Diagram of zebrafish vasculature. The orange line indicates site of measurement. (**C**) Vasculature of control and (**C**’) vasopressin-treated embryos at 48 hpf. (**D**) Diameter of cardinal vein (CV), dorsal aorta (DA), and intersegmental vessels (ISV). For D: n = 11 for wildtype, n = 11 for vasopressin-treated embryos. Two-sided *t*-test was performed to validate statistical significance; * indicates *p* < 0.05. Scale bar represents 50 µm.

In order to verify that vasopressin receptors are expressed embryonically in zebrafish, we performed reverse transcriptase PCR on the RNA extracted from either the dissected heart tissue or the whole embryos at 24, 48, or 72 hpf (Figure 1A and Figure S1B). At all of the timepoints tested, the *avtr_1a1_*, *avtr_1a2_*, and *avpr2* receptors were all expressed in the whole-body samples but not in the cardiac samples. The vasopressin receptors *avpr2aa, avpr2ab,* and *avpr2* were not detected in the cDNA made from whole zebrafish embryos (Supplemental Figure S1A). This finding implies that the observed cardiac phenotypes caused by vasopressin application do not arise from direct action of vasopressin on cardiac tissue, but rather reflect biomechanical impacts arising from changes in cardiac afterload and its downstream consequences.

In order to measure directly whether vasopressin elicits vasoconstriction in zebrafish embryos, we transferred Tg(fli1:EGFP) embryos at 24 h post-fertilization (hpf) to media containing 10 µM of vasopressin, and measured vessel diameters at 52 hpf by assessing the outside borders of the GFP-labeled endothelial cells (Figure 1B–D). We found that 24 h of vasopressin exposure was sufficient to cause significant vasoconstriction of the major blood vessels (18% constriction in the dorsal aorta and 25% constriction in the cardinal vein; *p* = 0.007 for dorsal aorta, *p* = 0.0004 for cardinal vein). Vessel radius is the major regulator of vascular resistance in the body (Resistance=(8μL)/(πr^4^); where µ is the fluid viscosity, *L* is the length of the vessel, and *r* is the radius of the vessel). Thus, an 18 or 25 percent constriction in diameter corresponds to a 124% and 281% increase in resistance, respectively, with the assumptions of Hagen–Poiseuille flow. Vasopressin application did not cause any detectable constriction of the intersegmental vessels (ISVs) (*p* = 0.43). In order to determine the minimal length of vasopressin exposure sufficient for significant vasoconstriction, we treated 24 hpf embryos with vasopressin for shorter periods and again measured the diameter of the dorsal aorta. The embryos that were exposed to 4 h of vasopressin treatment already showed significant constriction in their dorsal aorta (Supplemental Figure S1B). In order to determine how the length of vasopressin exposure induced constriction, we treated 24 hpf embryos with varying concentrations of vasopressin and then evaluated dorsal aorta diameter at 72 hpf (Supplemental Figure S2C). We found that treatment with 10 µM vasopressin caused a 50% decrease in dorsal aorta diameter at 72 hpf. The application of increasing concentrations did not produce greater constriction (data not shown). Among treated and untreated embryos, very few exhibited general abnormalities such as hemorrhaging, or body patterning defects (<5 out of 78 embryos scored presented either defect). In order to assess the degree of cell death, 72 hpf embryos were treated with acridine orange, a vital dye. The vasopressin-treated embryos did not exhibit significantly more cell death than the buffer-treated controls, suggesting that vasopressin treatment is not toxic to cells (Supplemental Figure S2A). Together, these data establish vasopressin exposure as a reliable method to increase afterload in zebrafish embryos without directly acting on cardiac tissue.

### 3.2. Increased Afterload Alters Cardiac Function and Growth

We next tested the hypothesis that increased afterload impairs zebrafish embryonic cardiac function. We exposed embryos to 0, 2.5, 5, 7.5, and 10 µM doses of vasopressin beginning at 24 hpf. After 16 h of continuous vasopressin exposure (i.e., in 40 hpf embryos), we found that increasing concentrations of vasopressin produced a dose-dependent effect on vessel constriction (Supplemental Figure S3), and therefore a dose-dependent effect on afterload. Doses of 5 µM or higher produced significant vessel constriction. In order to measure cardiac output, control and vasopressin-treated embryos were recorded by high-speed video. Kymograph analysis provided a measure of the velocity of red blood cells, which was then used to calculate flow rate. The parameters of cardiac function were computed from flow rate as previously described [30]. 

At 24 hpf, the heart has just formed a linear tube and is beginning to contract, but circulation is not yet robust. We measured the heart rate of embryos immediately before exposure to 10 µM vasopressin, and then after 5 min of vasopressin exposure (Supplementary Movie 1). There was no significant difference in heart rate post vasopressin exposure (Supplementary Figure S2B, *p* = 0.29, n = three control and three vasopressin-treated embryos). No overt change in morphology was noted. Consequently, changes noted at later time points are likely due to remodeling events and not a consequence of vasopressin application.

**Figure 2:**
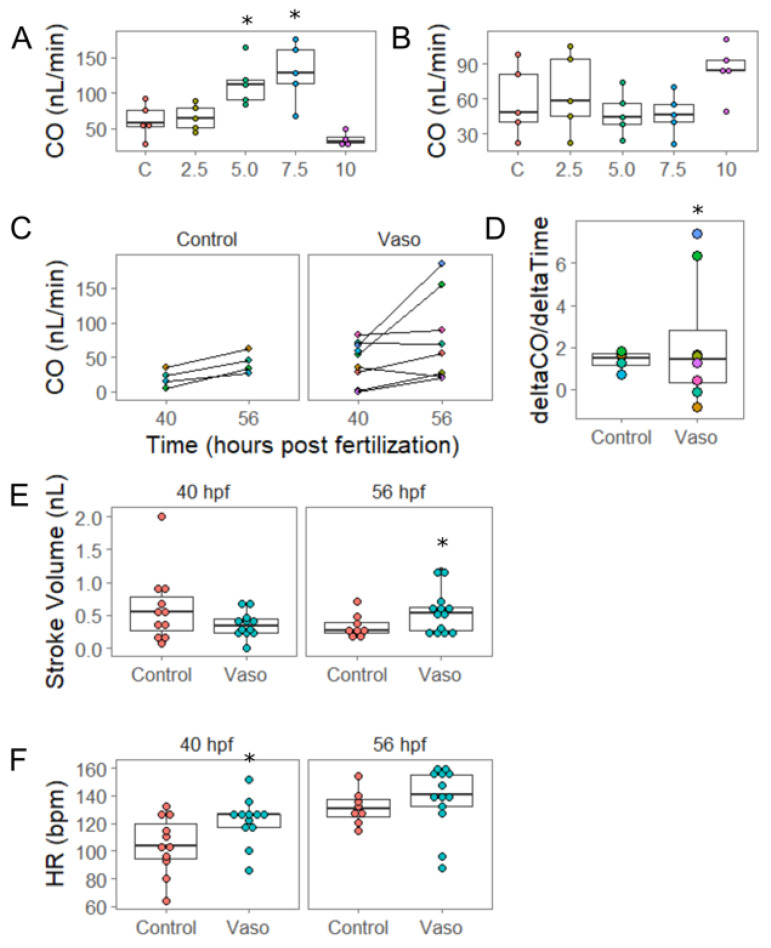
Increased pressure causes chamber remodeling by 56 hpf. (**A**) Cardiac output (CO) at 40 hpf at increasing doses of vasopressin (control, 2.5, 5, 7.5, and 10 µm). (**B**) CO at 56 hpf at increasing doses of vasopressin (vaso). n = 5 fish per group. (**C**) Cardiac output at 40 and 56 hpf in control and 10 µm vaso-treated fish. Each colored dot represents an individual fish; orange-colored dots represent control fish, light blue represents mildly-affected fish, and purple represents severely-affected fish. n= 4 control, 8 vaso. (**D**) Rate of change for cardiac output with respect to time. (**E**) Stroke volume and (**F**) heart rate of control and 10 µm vaso-treated fish at 40 and 56 hpf. n = 11 control and 12 vaso-treated fish at 40 hpf, and 8 and 13 vasopressin-treated fish at 56 hpf. * indicates *p* < 0.05.

At 40 hpf, the constriction at the AVJ has developed, but endocardial cushion formation has not yet initiated. The analysis of embryos at this time point revealed that embryos treated with 5 µM vasopressin produced a 1.85-fold increase in cardiac output, and embryos treated with the 7.5 µM dose produced a 2.11-fold increase in cardiac output relative to controls (Figure 2A, *p* = 0.03 for 5 µM, and *p* = 0.005 for 7.5 µM). These data clearly suggest that the hearts are sensing the change in load and are implementing functional responses to meet physiological need. Given that cardiac output is the product of stroke volume times heart rate, we investigated whether either of these measures changed. The stroke volume for embryos treated with either the 5 µM or 7.5 µM dose averaged approximately 33% larger than controls, providing some explanation for the increased cardiac output, though this difference was not statistically significant (Supplemental Figure S4, *p* = 0.25 for 5 µM, 0.07 for 7.5 µM). However, embryos at all doses demonstrated significant increases in heart rate equivalent to 125–135% of the controls, providing a further explanation for the increased cardiac output (Supplemental Figure S5, *p* = 0.04 for 2.5 µM, *p* = 0.007 for 5 µM, *p* = 0.003 for 7.5 µM, *p* = 0.04 for 10 µM). Together, these data taken at 40 hpf suggest that moderate increases in afterload (5 and 7.5 µM doses) triggered a compensatory response of increased heart rate, resulting in an increased cardiac output.

The embryos that were exposed to the 10 µM dose reacted differently than those exposed to lower doses. First, they did not achieve an increase in cardiac output by 40 hpf, but instead demonstrated a significant decrease in cardiac output (Figure 2C; control = 50.6 nL/min, vasopressin = 32.26 nL/min, *p* = 0.04 by *t*-test, n = 15 control, and 23 vasopressin-treated embryos). A subset of the embryos treated with 10 µM vasopressin (7 out of 23 embryos) exhibited disproportionate chambers and increased looping angle (Supplementary Movie 2). These embryos were termed “severely affected” while embryos that did not exhibit these overt morphological defects were termed “mildly affected”. The looping angle was evaluated by measuring the angle between the line bisecting the atrium and the line parallel to blood flow in the AVJ [30]. The control and mildly-affected embryos displayed similar looping angles (109° for control, 104° for mildly affected; n = 16 control, 16 mildly affected, *p* = 0.52 by Dunnett’s test), while severely-affected embryos had an average looping angle of 139° (n = 7, *p* = 0.002 against control by Dunnett’s test, Supplemental Figure S6A). 

In order to evaluate if treated chambers grew in proportion to those in the control hearts, we measured the cross-sectional area of the ventricle and atrium at the end of ventricular contraction and calculated the ratio of ventricular area to atrial area. Control and mildly-affected embryos displayed similar chamber area ratios (control = 0.42, mild = 0.47, *p* = 0.25 by Dunnett’s test), whereas the chamber area ratio of severely-affected embryos was significantly smaller (ratio = 0.27, *p* = 0.00013 by Dunnett’s test, Supplemental Figure S6B). In order to further analyze these data, we plotted embryo count against the looping angle or chamber area ratio (Supplemental Figure S7). In both cases, the severely-affected embryos clustered together, and the data distributions appeared bimodal (Hartigan’s dip test for unimodality, *p* = 0.03 for looping angle, and *p* = 0.05 for chamber area ratio). Together, these data indicate that under conditions of high afterload, about two-thirds of hearts develop the correct chamber morphology and looping angle by 40 hpf, while approximately one-third of hearts were compromised.

In order to understand how increasing afterload alters overall contraction patterns in the heart, we measured the speed of the progressing contractile wave in the control and the 10 µM vasopressin-treated embryos at 40 hpf. In this assay, we tracked successive points of maximal chamber lumen constriction as contractile wave progressed across the length of the heart tube. The speed of the contraction wave across the cardiac cycle was plotted against normalized time; where 0 corresponds to the onset of contraction at the atrial inlet, and one corresponds to the completion of ventricular systole. In the control embryos, the speed of contraction during atrial systole peaked at 2.4 mm/s, then slowed to 0.3 mm/s as the wave passed into the AVJ, followed by increases in speed as contraction proceeded through the ventricle (Supplemental Figure S8). In the 10 µM vasopressin-treated group, both mild embryos and severe embryos displayed similar contraction wave patterns: contraction through at the atrial inlet began with a similar speed (control average: 2.2 mm/s, vasopressin average: 2.5 mm/s, *p* = 0.30), but the speed did not peak near the end of atrial systole as occurred in the control embryos. Furthermore, the speed of the contraction wave in the AVJ itself was two to three-fold higher in both the mild and the severe vasopressin-treated embryos, a significant difference (Supplemental Figure S9A, n = 11 control, 15 mild, and 7 severe vasopressin-treated embryos; *p* = 0.03 for control vs. mild, and *p* = 0.003 for control vs. severe by Dunnett’s test). In order to correct for possible variability in the overall contraction speed in each heart, we normalized the AVJ speed to the maximum speed in each heart, but significant increases were still detectable (Supplemental Figure S9B). Together these data indicate that increased afterload has already altered cardiac contraction patterns by 40 hpf and specifically alters AVJ function, even in the mildly-affected group. 

In order to investigate how increasing afterload alters embryonic pumping mechanics, we evaluated the length of endothelial closure in 40 hpf embryos. During each cardiac cycle, there exists a period during which the endothelial layers lining either side of the AVJ come into brief physical contact. In the control embryos, the length of endothelial closure (i.e., the extent of maximal endothelial contact in the AVJ region) approximates 77 µm at the end of ventricular systole (see Supplemental Figure S10). Previously, our group identified the length of endothelial closure as a parameter that embryonic hearts can alter to adapt to increases in pressure and limit reverse flow [32]. Thus, we hypothesized that as afterload increased in relation to greater vasopressin concentrations, the length of endothelial closure would also increase. We measured the length of endothelial closure for each dose of vasopressin (from 0 to 10 µM). As predicted, vasopressin elicited a dose-responsive increase in the length of endothelial closure by 40 hpf (Supplemental Figure S10). These data suggest that hearts which are subjected to sustained high afterload use increased endothelial closure as one means to adapt to stressful pr.essure. 

By 56 hpf, the chambers have undergone primary ballooning and endocardial cushion development is robust. After 32 h of continuous vasopressin exposure (i.e., in 56 hpf embryos), we re-examined heart function to determine how well hearts had adapted to a period of sustained high afterload. Remarkably, by 56 hpf no significant differences in cardiac output were detected at any of the doses tested except for the 10 µM, which displayed significantly increased cardiac output (Figure 2C, *p* = 0.04 by Tukey’s). No embryos overtly exhibited weakened contractility. Since these findings contrasted with those of 40 hpf, we hypothesized that embryo hearts have adapted to rescue cardiac function by 56 hpf. In order to test this hypothesis, we tracked the individual control or 10 µM vasopressin-treated embryos and assessed cardiac function at both 40 and 56 hpf. We obtained time-matched data sets for four control and eight vasopressin-treated embryos, of which two had exhibited the severe phenotype at 40 hpf. Intriguingly, these two embryos did not display any overt morphological defects at 56 hpf (Supplementary Movie 3). The control embryos uniformly increased cardiac output between 40 and 56 hpf (n = 4), while the vasopressin-treated embryos exhibited much more variability in their response (n = 8; Figure 2C). The quantification of the rate of change using Bartlett’s test for homogeneity of variance confirmed that vasopressin-treated embryos responded with significantly more variability than the controls (Figure 2D; *p* = 0.01), though their overall rate of change was not significantly different (*p* = 0.27). These data indicate that at the highest tested condition of afterload, rather than continuing the downward trend in function seen at 40 hpf, embryos were on average able to sustain their CO by 56 hpf, and in some cases increase it over the controls. 

To better understand how the embryos at the 10 µM dose had modulated cardiac output, we analyzed stroke volume and heart rate at 56 hpf. No significant differences were detected in stroke volume at 40 hpf (*p* = 0.32 by Kruskal-Wallis). However, at 56 hpf, stroke volume was ~50% higher in the vasopressin-treated embryos compared to the controls, a significant change (Figure 2E, *p* = 0.04 by *t*-test). At 40 hpf, the vasopressin-treated embryos had demonstrated heart rates approximately 7% higher than the controls, a significant change (Figure 2F, *p* = 0.03 by *t*-test), but heart rates at 56 hpf matched the controls. These data suggest that increased heart rate was the initial adaptation to compensate for increased afterload at 40 hpf, but that increased stroke volume was the primary driving force that maintained (or increased) cardiac output at 56 hpf. 

### 3.3. Increased Afterload Induces Cardiac Hypertrophy and Alters Sarcomere Abundance

Compared to the lower doses, the 10 µM treated embryos took longer to ramp up their response of increased cardiac output, as might be expected by the work needed for hearts to pump against such high resistance. We hypothesized that the hearts in 56 hpf treated embryos had structurally remodeled to provide a sustainable response. The next series of experiments focused on the 10 µM treated embryos in order to investigate this hypothesis. 

Murine models indicate that hypertrophy in the cardiac ventricle reflects an increase in myocardial cell size, while embryonic chicks undergo hyperplasia in response to conotruncal banding [33,34]. In order to evaluate whether or not zebrafish experience hyperplasia in response to increases in afterload, we exposed the Tg(myl:dsred-nuc) embryos, which express dsRED in the nucleus of cardiomyocytes, to 10 µM vasopressin from 24 to 56 hpf and evaluated the ventricular cell number. We found no change in the number of ventricular cardiomyocytes between the control and the vasopressin-treated embryos (control average = 82, vasopressin average = 87, *p* = 0.48, n = four control and five vasopressin-treated embryos, Supplementary Figure S11). In order to evaluate ventricle size in zebrafish, the Tg(myl7:eGFP) embryos, which express GFP in cardiomyocytes [35], were treated with 10 µM vasopressin from 24–56 hpf. The vasopressin-treated hearts showed a significant increase in overall ventricular volume (Supplemental Figure S12). In order to further determine if increases in loading increased the myocardial cell size or shape, we evaluated cardiomyocytes using rhodamine-labeled phalloidin, which binds actin fibers located predominantly near the cell periphery. By 52 hpf, myocardial cells in the mid-ventricle were 17% larger in the vasopressin-treated hearts relative to the controls (*p* = 0.04), but retained a similar degree of circularity (Supplemental Figure S12; *p* = 0.23). Thus, application of vasopressin to zebrafish embryos recapitulates the hypertrophic effects of afterload observed in other vertebrate animal models.

Because embryos exposed to the 10 µM dose of vasopressin experienced an increase in stroke volume, we hypothesized that they may increase their myofibril content on a per-cell basis. Following the approach of Lin et al. (2012), we used the Tg(myl7:cypher-eGFP) transgenic line to visualize z-bands in the heart, and quantify their size and number per cell [36]. The summed length of all z-bands represents the total z-band size (TZB) per cell (Supplemental Figure S13. The TZB is normalized to the planar cross-sectional area of the cell to assess the total myofibril content per cell while accounting for differences in cell size.

**Figure 3:**
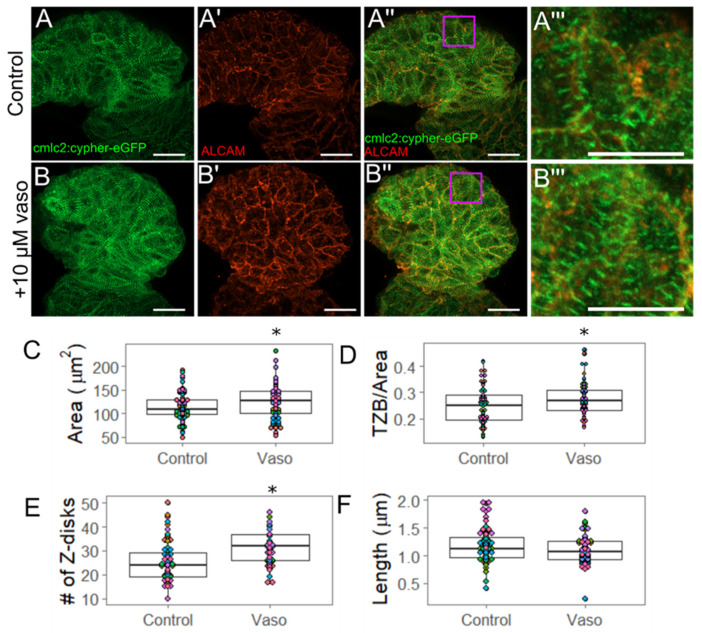
Increased pressure causes hypertrophy of ventricular outer curvature (OC) myocytes by 56 hpf. (**A**) Control and (**B**) vaso-treated hearts showing z-disks (**A**,**B**) and cell shape through ALCAM immunohistochemistry (**A**′,**B**′). (**A**′′,**B**′′) display merged images. Purple box shows regions of interest shown in (**A**′′′,**B**′′′). For **A**–**A**′′, and **B**–**B**′′, scale bar represents 20 µm. (**A**′′′) Control and (**B**′′′) vaso-treated hearts. Scale bar represents 10 µm. (**C**) Area of ventricular OC cells in control and vaso-treated hearts. (**D**) Total z-disk size (TZB) of sarcomeres present in each cell normalized to the area of each cell (1/µm). For details for how TML was measured, see supplemental Figure S11. (**E**) Number of z-disks per cell in control and vaso-treated hearts. (**F**) Average length of the sarcomeres present in each cell. For control and vaso-treated hearts, 14 fish were analyzed. Five cells from the OC were analyzed per fish, such that 70 total cells were analyzed. Each colored dot on the graph above represents an individual fish. * indicates *p* < 0.05, Student’s *t*-test was used for statistical comparisons.

In order to determine whether increased myofibril content could account for the increase in stroke volume observed in embryos treated with the 10 µM dose of vasopressin at 56 hpf, we measured myofibril content of individual ventricular outer curvature cardiomyocytes using the Tg(myl7:cypher-eGFP) line, in which cypher (a z-disc protein) has been tagged with eGFP (Figure 3). We performed immunohistochemistry using the ALCAM (activated leukocyte cell adhesion molecule; also called DM-GRASP) antibody, which marks myocardial cell borders to measure the cross-sectional area of each ALCAM-labeled cell (Figure 3A,B’’’). We observed that cells exposed to 10 µM vasopressin were approximately 10% larger than their untreated counterparts (*p* = 0.04) (Figure 3C), consistent with the rhodamine-phalloidin data above. After being normalized to the individual cell area, the data indicated an approximately 5% average increase in TZB in vasopressin-treated cardiomyocytes beyond what was expected by the cells’ larger size (*p* = 0.03) (Figure 3D). Further experiments were conducted to determine whether the z-band number (a rubric for the number of sarcomeres present) or average z-band size (reflecting the thickness of the sarcomere) accounted for the increased TZB. We found the number of z-bands per cell was significantly increased from 25.3 z-bands/cell in the control embryos to 32.4 z-bands/cell in the vasopressin-treated embryos (*p* < 0.001) (Figure 3E), suggesting that treated cells increased their myofibril content. However, the average length of individual z-bands was not significantly affected (*p* = 0.24) (Figure 3E), suggesting that myofibril thickness was not changed. This pattern of increased number of sarcomeres is consistent with pathological concentric hypertrophy. 

### 3.4. Increased Afterload Induces Retrograde Flow and Alters AVJ Mechanics

**Figure 4:**
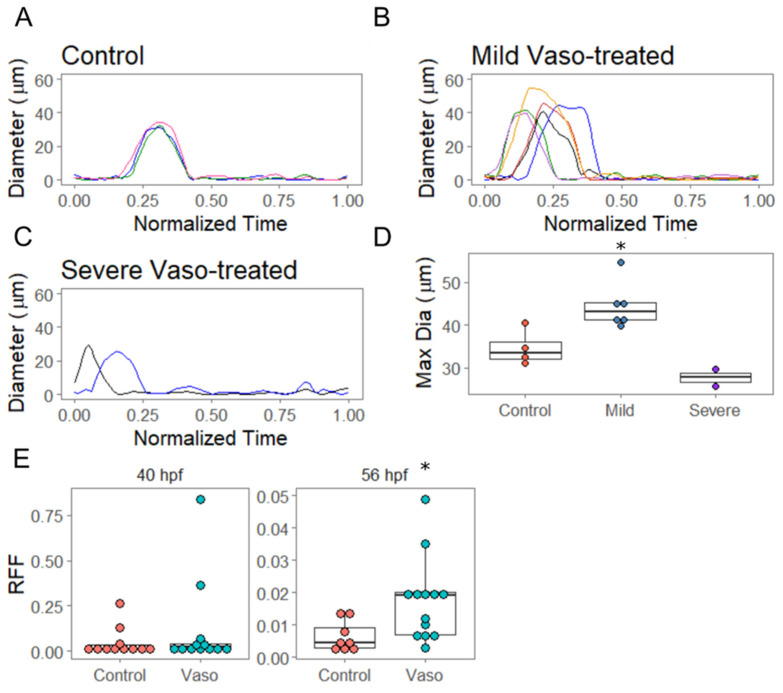
Increased afterload increases retrograde flow and alters AVJ mechanics. (**A**) Diameter of the flow field at the atrioventricular junction in control hearts at 56 hpf. *X*–axis is normalized time across a cardiac cycle, where 0 corresponds to the beginning of atrial contraction and 1 represents the end of ventricular contraction. n = 4 (**B**) Diameter analysis of vaso-treated hearts that showed hypertrophic growth between 40 and 56 hpf. n = 6 (**C**) Diameter analysis of vaso-treated hearts at 56 hpf. These hearts showed severely altered pumping mechanics at 40 hpf. (**D**) Maximum diameter analysis of control, vaso-treated hearts that showed altered pumping (AP) and vaso-treated hearts that showed hypertrophic growth (HH). (**E**) Retrograde flow fraction (RFF) at 40 and 56 hpf in control and vaso-treated hearts. n = 11 control and 12 vaso-treated fish. * indicates *p* < 0.05.

In order to evaluate the valve formation and function, we measured how the diameter of the AVJ varied across the cardiac cycle in the hearts of live embryos. The hearts of the vasopressin-treated embryos were scored initially as either ‘mild’ or ‘severe’ based on their appearance at 40 hpf, and then videoed at 56 hpf. We noted that the dynamics of AVJ behavior during the cardiac cycle were significantly altered under conditions of increased loading (Figure 4). The AVJ of the control embryos displayed uniform behavior, opening with an average maximal diameter of 34.7 µm (Figure 4A,D). The vasopressin-treated embryos initially scored as ‘mild’ showed significantly increased average maximal diameter of 44.5 µm by 56 hpf (*p* = 0.015 by *t*-test) (Figure 4B,D). The vasopressin-treated embryos initially scored as ‘severe’ at 40 hpf displayed reduced average maximal diameters of 27.7 µm by 56 hpf (*p* = 0.10 by *t*-test) (Figure 4C,D). Moreover, AVJs in the vasopressin-treated hearts open significantly earlier than the control counterparts (controls at 40% all vasopressin-exposed embryos at 11%, *p* = 0.04). Both groups of vasopressin-treated embryos displayed an increased retrograde flow fraction (RFF), quantified as follows: $\int_{0}^{T}\left|Q_{reverse}\right|dt/\int_{0}^{T}\left|Q_{total}\right|dt$, where *Q* is the flow rate and *T* is the cardiac cycle period [32]. In the vasopressin-treated embryos, RFF increased approximately three-fold over controls by 56 hpf (Figure 4E, *p* = 0.01). Together, these data demonstrate that despite the ability of the 10 µM vasopressin-treated hearts to maintain cardiac output, the alterations in loading nevertheless substantially impacted other aspects of heart development, including blood flow patterns and AVJ closing dynamics. 

### 3.5. Increased Afterload Alters Myocardial AVJ Cell Specification

**Figure 5:**
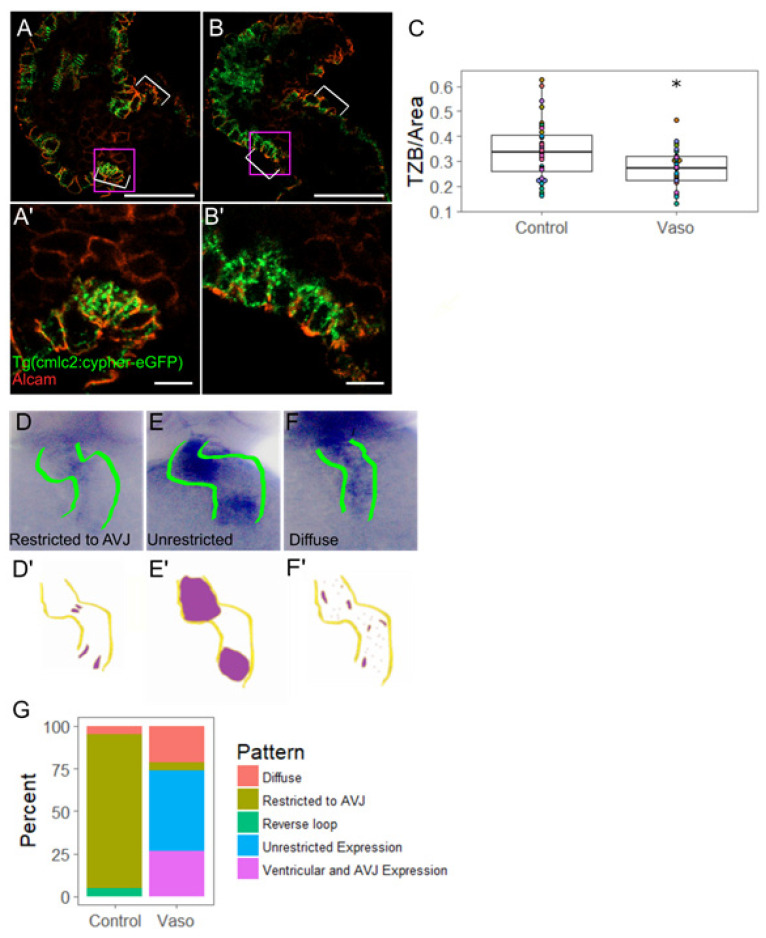
Increased pressure alters AVJ myocyte morphology and specification. (**A**) Control and (**B**) vaso-treated hearts showing z-disks and cell shape through ALCAM immunohistochemistry at 56 hpf. Purple box shows region of interest shown in (**A**′,**B**′). For (**A**,**B**), scale bar represents 20 µm. (**A**′) Control and (**B**′) vaso-treated hearts. Scale bar represents 10 µm. White arrow heads indicate AVJ. (**C**) TZB of sarcomeres present in each cell normalized to the area of each cell (1/µm). n = 36 cells for control and 33 for vaso-treated hearts. (**D**) In situ hybridization (ISH) of myocardial AVJ marker bmp4 in control and (**E**,**F**) vaso-treated hearts. (**D**′–**F**′) Diagrams of ISH expression patterns. (**G**) Percent of fish expressing a particular bmp4 expression pattern. A total of 20 fish were scored in each group. * indicates *p* < 0.05.

Given that AVJ functional dynamics were altered in conditions of increased afterload, we next explored whether differentiation of AVJ myocardial, and/or endocardial cells, were affected in conditions of high afterload. Focusing first on myocardial differentiation specifically at the AVJ, we evaluated the cellular morphology based on ALCAM immunostaining. By 48 hpf, wildtype AVJ myocytes have acquired a distinct trapezoidal cell morphology, express Tbx2b, and direct endocardial cell specification via Bmp signaling [23,37]. In the vasopressin-treated embryos at 56 hpf, AVJ myocytes were on average more circular than their control counterparts (Supplemental Figure S14A, *p* = 0.03), suggesting that AVJ myocytes had not fully differentiated. In contrast to the ventricular outer curvature myocytes, which enlarge (Figure 3), the AVJ myocytes spanned approximately the same area in vasopressin-treated hearts as in the controls, suggesting that AVJ myocytes do not become hypertrophic (Supplemental Figure S14B *p* = 0.62). Upon assessing the total myofibril content, we observed a 22% decrease in normalized TZB in the vasopressin-treated AVJ myocytes (Figure 5C, n = 11 control, 10 vasopressin-treated hearts, *p* = 0.003). 

Next, we assessed the specification of AVJ myocytes via in situ hybridization using *bmp4* and *tbx2b* probes [38]. In normal hearts, myocardial *bmp4* and *tbx2b* are broadly expressed in the ventricle at 33–36 hpf. During the second day of development, the wildtype hearts gradually down-regulate expression of these markers in the chambers while enriching them at the AVJ, such that AVJ-specific enrichment is evident by 52 hpf [39]. Failure of these markers to retract to the AVJ is observed in mutant or pharmacological conditions associated with abnormal valve development [38]. In approximately 68% of the vasopressin-treated hearts at 56 hpf, *bmp4* expression failed to retract to the AVJ and instead remained broadly expressed in the ventricle or diffusely expressed throughout the heart (Figure 5G). In contrast, *tbx2b* expression was not notably altered by vasopressin-treatment (fold change = 1.10, *p* = 0.42 by qPCR on dissected hearts). Taken together, these data demonstrate that cardiac afterload produces a biomechanical stress that precludes normal cell differentiation of myocardial cells in the AVJ. 

### 3.6. Increased Afterload Alters Endocardial AVJ Cell Specification

**Figure 6:**
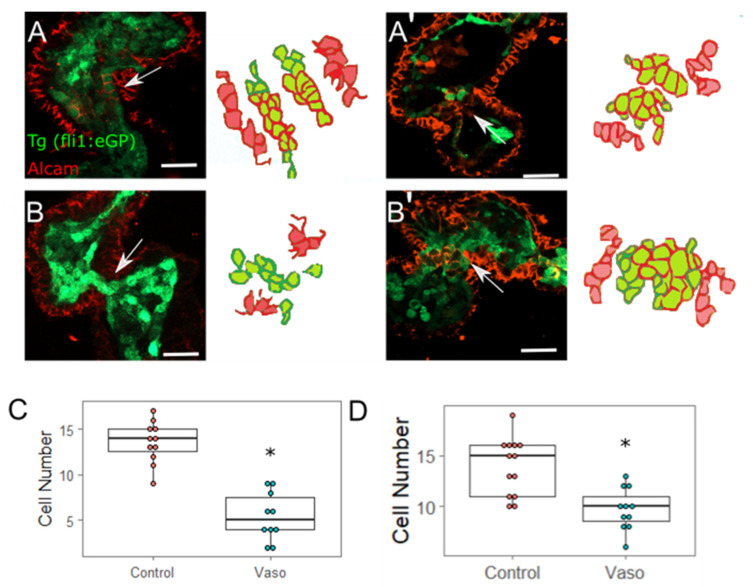
Increased myocardial afterload causes aberrant AVJ cell specification. (**A**) Control hearts stained with ALCAM at 56 hpf. (**A**’) Control hearts stained with ALCAM at 72 hpf. Scale bar represents 20µm. (**B**,**B**’) Representative image of vaso-treated heart at (**B**) 56 hpf and at (**B**’) 72 hpf. (**C**) Number of ALCAM expressing endocardial cells. N = 11 wildtype hearts, 10 vaso treated hearts at 56 hpf. Statistical significance was determined using Student’s *t*-test; * indicates *p* < 0.05. (**C**,**D**) Number of ALCAM expressing endocardial cells. N = 11 wildtype hearts, 10 vaso-treated hearts at 72 hpf. Five cells were analyzed per location per heart, area was measured for each cell and averaged. AVJ myocardial cells were defined as myocardial cells located in the constriction between the atrium and ventricle.

During valve development in mammals, endocardial cells undergo EMT, invade the endocardial cushions and become specified valve cells [40]. Although controversy exists regarding whether true EMT occurs in the AVJ of zebrafish [41,42], AVJ endocardial cells transition to a more cuboidal shape by 48 hpf, and endocardial cells from the superior valve leaflet fold into the cardiac jelly around 60 hpf. ALCAM is a well-characterized marker that appears at the cell periphery from 52 hpf onward only in AVJ endocardial cells that have become specified to a valve cell fate [43] (in contrast, myocardial cells express ALCAM throughout development). In order to evaluate endocardial AVJ development, we performed immunohistochemistry on the dissected wildtype and vasopressin-treated hearts at 56 hpf to detect ALCAM. Since these hearts were derived from Tg(fli1:eGFP) embryos, this method labels myocardial cells (red), chamber endocardial cells (green), and nascent valve endocardial cells in the AVJ (double-labeled red and green) (Figure 6A,B’). The results indicated a 45% reduction in the number of double-labeled ALCAM-positive valve-forming endocardial cells in the vasopressin-treated embryos (Figure 6C, *p* = 0.000019). In order to test whether AVJ formation was simply delayed in the vasopressin-treated embryos, we repeated the same experiment at 72 hpf. We again noted a significant reduction (approximately 30% fewer) in the number of ALCAM positive endocardial cells in the vasopressin-treated embryos (n = 12 control, 11 vasopressin treated; *p* = 0.004). These data strongly suggest that aberrant loading perturbs AVJ development by limiting the number of endocardial AVJ cells that achieve a specified state.

## 4. Discussion

Here we present vasopressin-treated zebrafish as a novel in vivo model suitable for the study of the impact of increased myocardial afterload upon the forming heart. Vasopressin treatment in embryonic zebrafish induces blood vessel constriction, leading to an altered biomechanical condition consistent with increased afterload. We found that high afterload produced a pathological cardiac phenotype, and that the hearts subjected to different vasopressin doses appeared to adapt in different ways. Specifically, the embryos treated with moderate doses of vasopressin (corresponding to moderate increases in afterload) responded by increasing cardiac output at 40 hpf, a change which allowed them to continue to function adequately at 56 hpf. The embryos treated with a high dose of vasopressin (corresponding to a higher increase in afterload) could not increase cardiac output, but were able to maintain it, initially by increasing heart rate (at 40 hpf) and later by increasing stroke volume (at 56 hpf). The hearts in roughly one-third of these high-dose embryos were clearly compromised at 40 hpf and failing at 56 hpf. However, the hearts in two-thirds of the high-dose embryos produced adaptive responses that augmented their ability to continue to function with a normal cardiac output at 56 hpf. Even in these hearts, cardiac pathology is evident by weaker contractility, slower contractile wave speeds, and increased variability in cardiac output. Evidence of putative adaptive responses include an expanded length of endothelial closure within individual heartbeats, induction of hypertrophy via increased cell size and myofibril content and increased cardiac output by altering heart rate or stroke volume. Within the AVJ, the negative impact of high afterload is evident by the disrupted AVJ differentiation and the increased fraction of retrograde flow. Though application of vasopressin produced variable responses, all treated hearts exhibited signs of pathology by 56 hpf. Given that the embryonic heart does not express vasopressin receptors itself, we conclude that vasopressin indirectly produces a high afterload, by altering the biomechanical environment, and that these changes are pathological for normal cardiac development. 

Prior studies using out-flow tract ligation to alter afterload in the chick embryos provided initial evidence to suggest that biomechanical signals derived from afterload impact function in the embryonic heart [6]. Limitations of this model include the requirement for time-consuming surgical manipulations and the limited amenability to genetic manipulation. The zebrafish vasopressin model complements and extends the chick ligation approach. The ease of vasopressin application enables high-throughput analysis of phenotypes and enables investigation of the timing and severity of responses to different doses. The existence of numerous characterized mutant lines in zebrafish offers the further potential to assess the impact of afterload under various genetic backgrounds. In order to thoroughly understand the molecular circuitry underlying the afterload-response, it will be necessary to couple manipulations in loading with manipulations in genetics.

This report provides the first detailed investigation of the functional, cellular, and molecular consequences of increasing afterload in the embryonic zebrafish heart. The zebrafish embryonic heart does not express vasopressin receptors, making it unlikely that vasopressin exerts any direct effects on cardiac contractility. Upon vasopressin treatment, vasoconstriction is detected in multiple vessels after as little as 4 h of exposure. The degree of constriction produced by 10 µM is calculated to generate at least a 124% increase in vascular resistance of the dorsal aorta, thus generating a robust increase in cardiac afterload. Increasing the load on the embryonic heart during early development (linear heart tube stage and beyond) dramatically altered cardiac function. In zebrafish, the heart rate of the 10 µM vasopressin-treated embryos increased initially at 40 hpf, but the increase was not maintained at 56 hpf. In contrast, stroke volume initially matched the controls at 40 hpf, but by 56 hpf had significantly increased. Concomitantly, all of the embryos exposed to 10 µM vasopressin showed a significant increase in myofibril content by 56 hpf. These data suggest that upon exposure to high afterload, embryos initially respond by increasing their heart rate, perhaps as a temporary compensatory measure. However, upon chronic exposure to high afterload, embryonic hearts remodel to meet the physiological demands. 

More specifically, high afterload produced hypertrophy of the ventricular OC cardiomyocytes by 56 hpf by mechanisms that increased ventricular cell size and increased myofibril content. This finding is consistent with studies of cultured human embryonic myocardial stem cells that displayed hypertrophy as well as increased proliferation when grown in conditions of high afterload [44]. Of note, high afterload in human adults can also lead to myocardial hypertrophy, reflecting increased ventricular cell size and increased myofibril content [45,46]. In contrast, chick embryos did not display cardiomyocyte hypertrophy under conditions of high load, but instead showed evidence of cardiomyocyte hyperplasia [34]. Which mechanotransductive mechanisms facilitate these changes remains an open question but may include disrupted endocardial-ECM-myocardial signaling, pressure-sensitive receptors, or flow-triggered endocardial responses then communicated to the myocardium. This finding suggests that high afterload either perturbs communication between the endocardium and the myocardium or generates biomechanical signals other than RFF and shear stress that impact cardiac hypertrophy. 

Strikingly, retrograde flow was significantly increased by 56 hpf in the vasopressin-treated embryos. Retrograde flow normally occurs in embryonic hearts aged ~36–72 hpf for a limited period of each heartbeat [30,32]. The movement of blood cells or plasma against the endocardial cells within the AVJ generates wall shear stress. Imaging studies have uncovered a required contribution of shear-stress related biomechanical forces for regulating normal cardiac valve development [42,47,48]. Vermot and colleagues established that retrograde flow (i.e., “reversing flows”) constitutes a unique physical stimulus for valve development, and showed that loss of RFF produced defects in endocardial cushion formation [47]. Moreover, the complex oscillatory flow patterns present in the AVJ prior to valve formation create shear-stress patterns that trigger endocardial cells to converge into the endocardial cushions [49]. In our case, increased RFF did not alter the number of EC converged at the AVJ by 56 hpf (Supplemental Figure S15), but we did observe that fewer of those ECs expressed the valve-specification marker, ALCAM (Figure 6). It is intriguing to speculate that distinct mechanosensitive mechanisms may regulate endocardial convergence in the AVJ and valve-cell differentiation. Of note, high afterload has the potential to impact the heart prior to the onset of detectable RFF (~36 hpf), and therefore may elicit defects independently of complex flow patterns [32,47].

The cellular mechanisms leading to reduced specification of endocardial AVJ cells remain unresolved. The myocardial cells at the AVJ secrete paracrine factors that promote endocardial valve cell specification. We speculate that an increase in loading during the vulnerable valve-formation period may interfere with the appropriate targeted secretion of key signaling factors by AV myocardial cells. Alternatively, inadequate reception of these signals by endocardial receptors may lead to fewer specified endocardial valve cells. Indeed, expression of the myocardial marker *bmp4* failed to become restricted to the AVJ by 56 hpf in the vasopressin-treated embryos, suggesting that myocardial AVJ cell specification is compromised in the vasopressin-treated embryos. In the myocardium, Bmp induces transcription factors critical for continued myocardial AVJ development, whereas in the endocardium, the Bmp–Wnt signaling axis promotes AVJ endocardial specification [50,51]. Thus, disruptions in Bmp signaling may be expected to perturb development in both myocardium and endocardium. 

Taken together, our AVJ data show that under conditions of high afterload, the AVJ myocardial cells fail to develop the myofibillar content expected for their developmental stage, fail to appropriately transition to the trapezoidal shape expected for differentiating AVJ myocardial cells, and fail to display the AVJ enriched pattern of myocardial markers indicative of maturing AVJ. We postulate that these defects in AVJ myocardial cell differentiation interfere with the normal communication between the endocardium and the myocardium and contribute to a valve defect. 

Many open questions remain regarding the mechanobiological means by which afterload elicits the pathological effects shown in this study. Firstly, are some of the high afterload-associated phenotypes mediated via alterations in shear stress or oscillatory flows, whereas others are mediated via mechonotransductive cues related to pressure? Prior work in chick models of high afterload suggest that high load can induce shear-responsive genes and dysregulation of the ECM. Similar mechanisms may be at play in vasopressin-treated embryos [19]. Secondly, how are afterload-mediated mechanical signals transduced into genetic signals? One intriguing hypothesis relates to the cell-stress response. Increased afterload increases ventricular wall stress. In vivo experiments using coronary artery ligation in adult rat models show that increasing wall stress causes remodeling of the t-tubule network and disrupts calcium homeostasis [52]. Interestingly, calcium transients have to been shown to regulate to Bmp signaling during *Drosophila melanogaster* neural development [53]. It is possible that increasing afterload alters calcium homeostasis during cardiac development, leading to altered Bmp signaling. More work needs to be done to investigate this hypothesis, as well as other potential cellular mechanisms for sensing and responding to afterload. 

## 5. Conclusions

Congenital valve defects arise from aberrations in valve formation during fetal development and frequently cause infant mortality. Surgery, often the only treatment available, has a ~28% mortality rate. Despite the high clinical burden, more work needs to be done to understand the underlying molecular and genetic causes of these defects. Here, we present a zebrafish model wherein high afterload is able to induce a valve development defect and postulate that afterload acts as a biomechanical cue that cells read and respond to in order to mediate valve development.

## Data Availability

Not applicable.

## Supplementary Materials

The following are available online at https://www.mdpi.com/article/10.3390/jcdd9010022/s1: Figures S1–S15 and Movies 1–3.
