# Supplementary material for: Myocardial Afterload Is a Key Biomechanical Regulator of Atrioventricular Myocyte Differentiation in Zebrafish
Source: J Cardiovasc Dev Dis. 2022 Jan 12;9(1):22. doi: 10.3390/jcdd9010022 (PMC8779957; doi:10.3390/jcdd9010022)
Supplement: Supplementary file 1 [file jcdd-09-00022-s001.zip › jcdd-1534391-supplementary.pdf]

SUPPLEMENTAL MATERIAL

Supplementary Figures

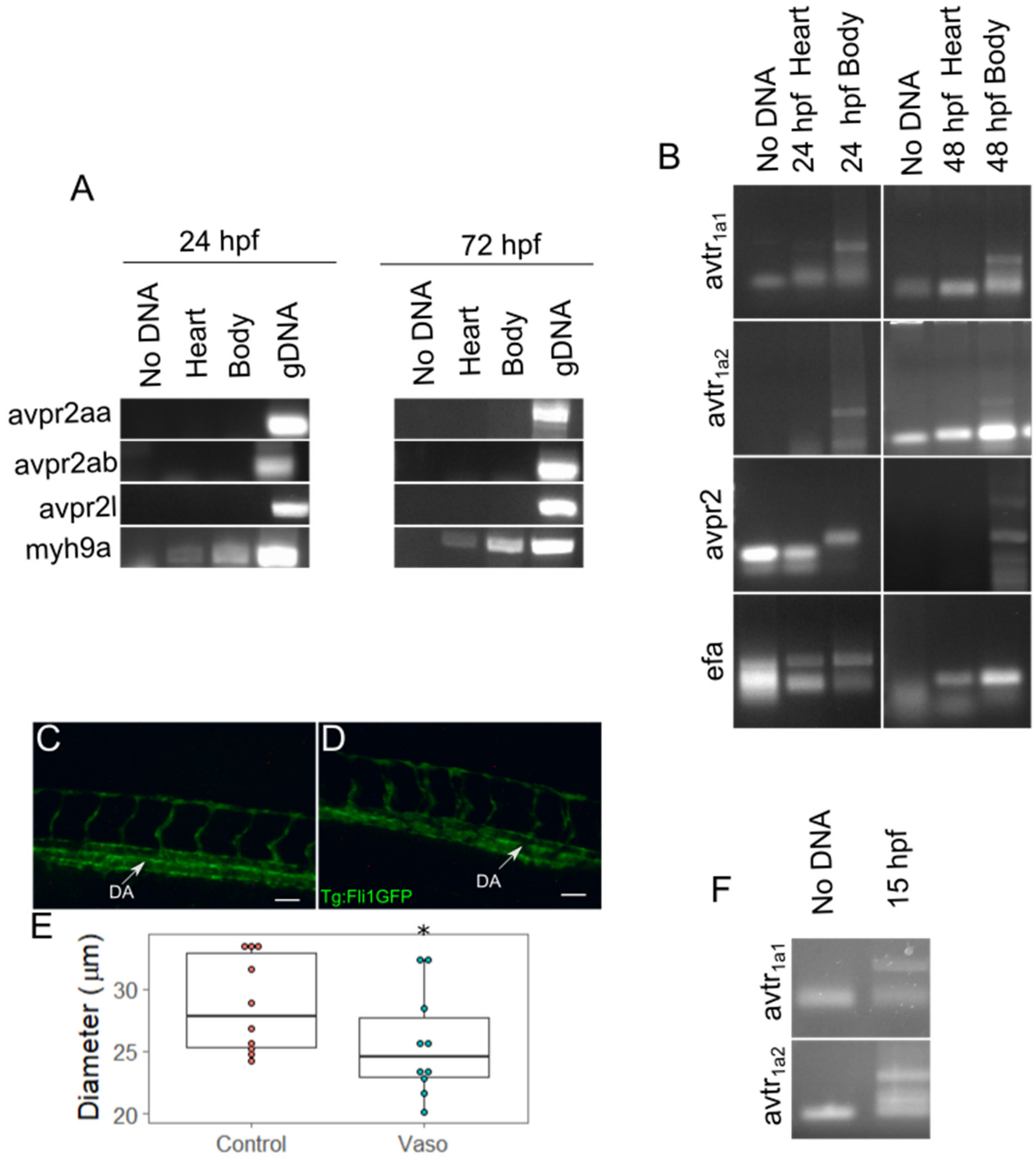

Supplemental Figure S1. Vasopressin application causes constriction in as little as 4 hours post application. A) Vasopressin receptors avpr2aa, avpr2ab, and avpr2l are not expressed in tissue either at 24 hpf, or at 72 hpf, though the PCR was able to amplify each gene from genomic DNA. Control gene *myh9a* is robustly expressed in all DNA samples B) Vasopressin receptors are not expressed in cardiac tissue either at 24 hpf, or at 48 hpf, in contrast to whole bodies. Control gene *efa* is robustly expressed in all DNA samples. B) Control vessels at 28 hpf. See figure 1 for expected band sizes. C) Vasopressin-treated vessels at 28 hpf. Vasopressin was applied at 24 hpf. D) Diameter of the dorsal aorta in vasopressin-treated embryos is significantly smaller than in control embryos. Error bars

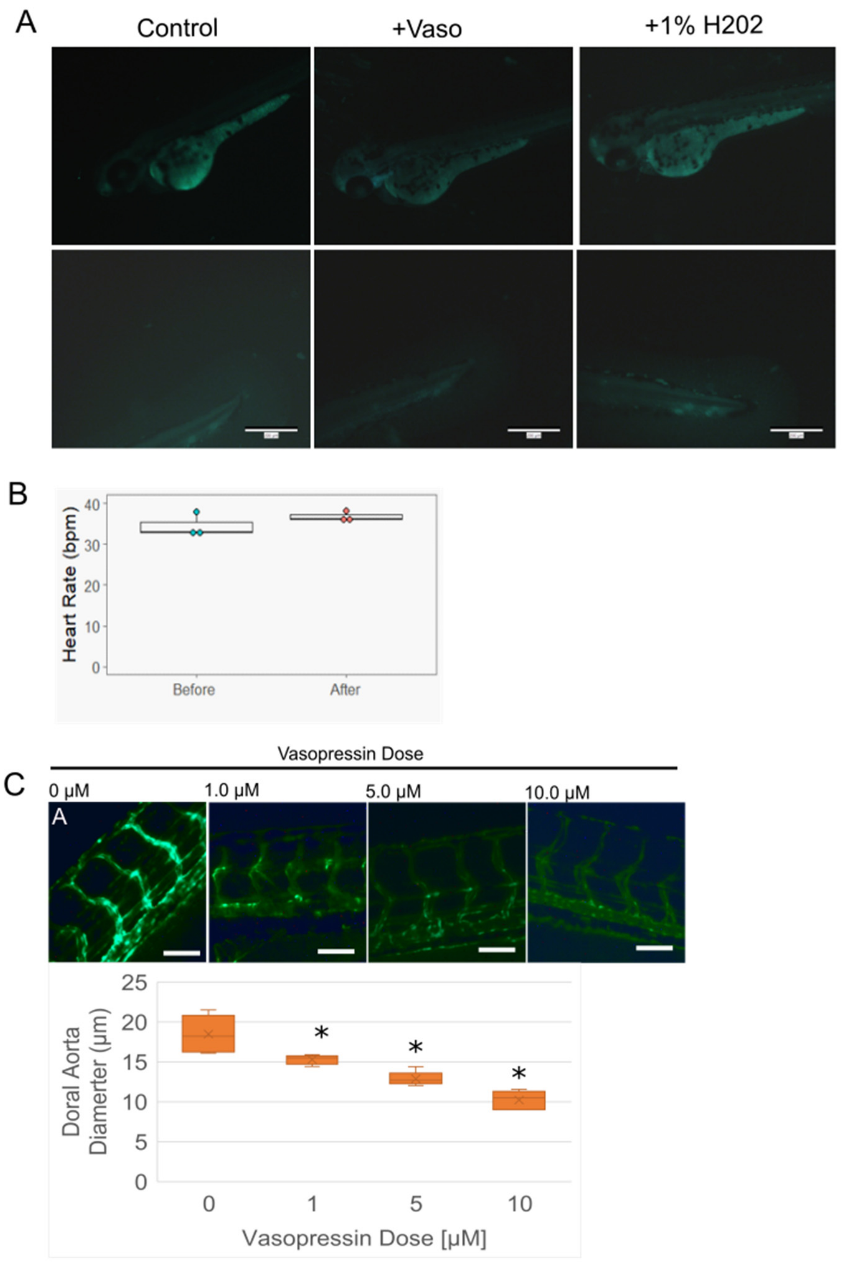

Supplemental Figure S2: A) asopressin application does not detectably cause cell death by 72hpf. Acridine orange stain was applied to detect cell death in embryos. Control and vasopressin-treated embryos have no significant signal other than the yolk autofluorescence, while positive control embryos treated with 1% H_2O_2 show foci throughout (indicated by the white arrows). Foci indicate cell death. B) Vasopressin exposure does not result in immediate changes in cardiac function. After 5 minutes of vasopressin-exposure, heart rates of 24hpf embryos did not change. N = 3 control, and 3 vaso-treated embryos. P = 0.29 C) Vasopressin application results in a dose dependent increase in constriction through 72 hpf. N= 5 embryos measure per dose, p= 0.02 for control vs 1µM, p= 0.001 for control vs 5µM, p= 0.0001 for control vs 10 µM.

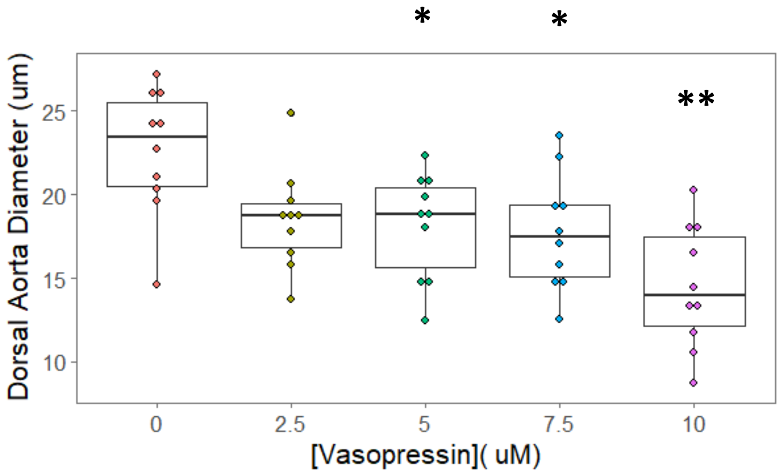

Supplemental Figure S3: Vasopressin application induces a dose-dependent constriction on the zebrafish dorsal aorta by 40hpf. Statistical significance was determined using Anova. N= 10 embryos were analyzed for each dose. Control vs 5 μM, p = 0.04; control vs 7.5 μM, p = 0.02; control vs 10 μM, p < 0.0001 .

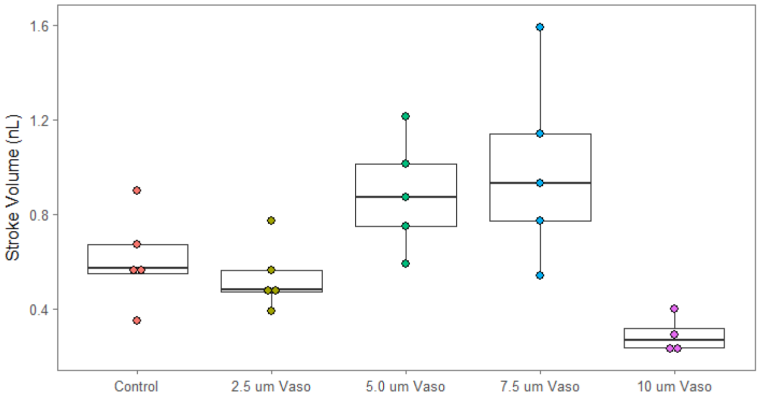

Supplemental Figure S4: Increased afterload does not significantly alter stroke volume; however, hearts under extremely high afterload trend toward decreased stroke volume. Dunnet's test was used to assess statistical significance. N=5 fish analyzed per treatment. Control vs 2.5 μM, p = 0.93; control vs 5 μM, p = 0.25; control vs 7.5 μM, p = 0.07; control vs 10 μM, p = 0.19

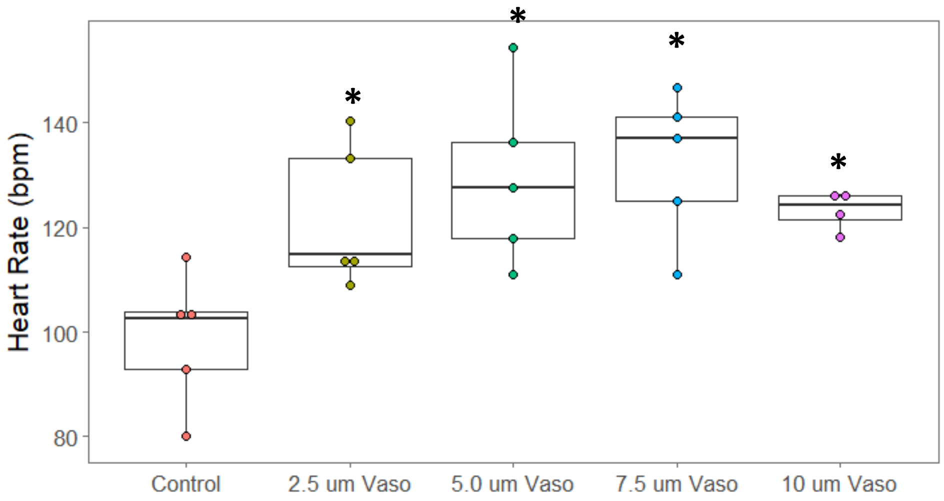

Supplemental Figure S5: Increasing concentrations of vasopressin produce increases in heart rate at 40 hpf. Dunnett's test was used to assess statistical significance. * indicates $p<0.05$. N=5 fish analyzed per treatment. Control vs 2.5 μM, p = 0.04; control vs 5 μM, p = 0.007; control vs 7.5 μM, p = 0.003; control vs 10 μM, p = 0.04.

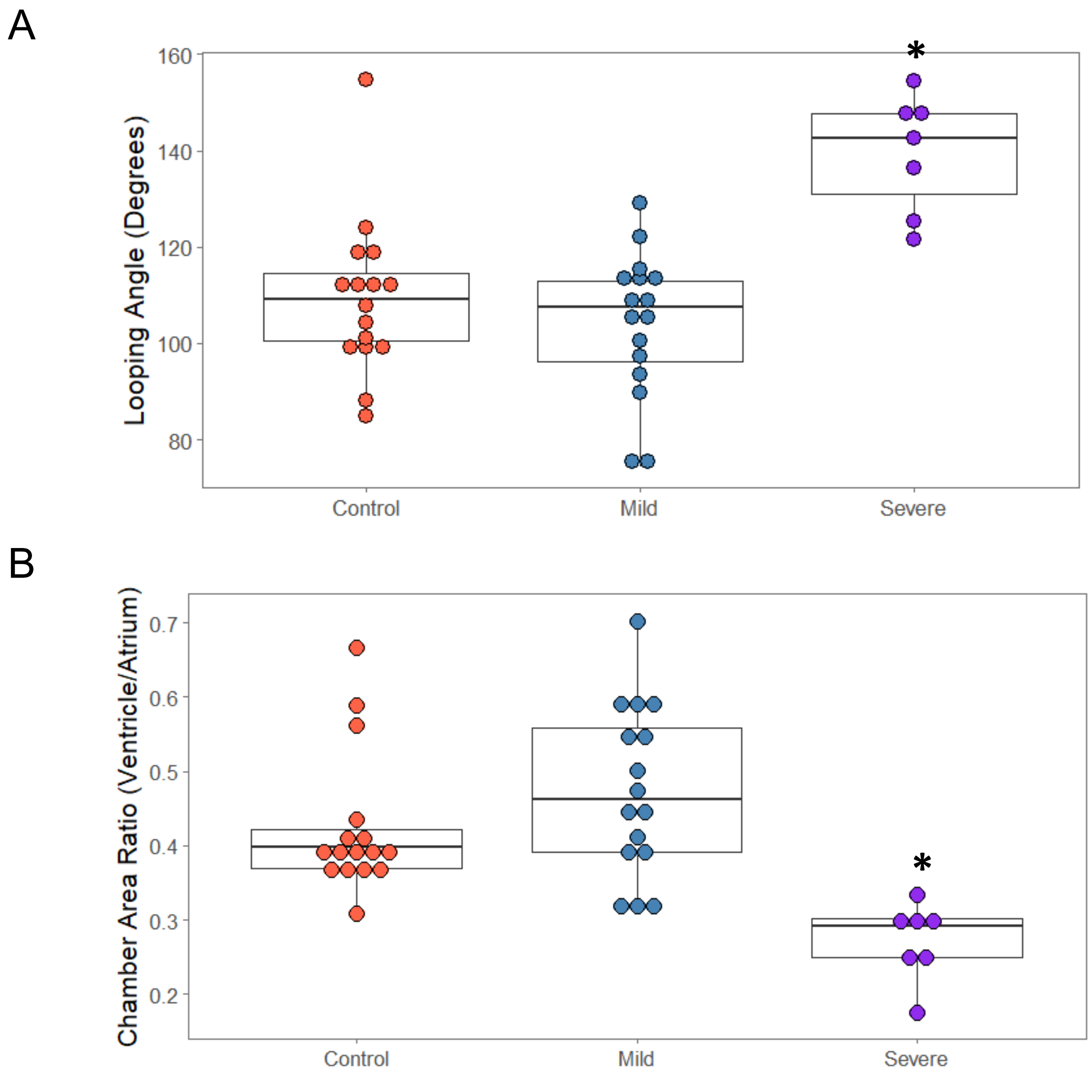

Supplemental Figure S6: A portion of the 10μM vasopressin-treated embryos demonstrate overt morphological defects. A) Looping angle is significantly increased in the “severe” group, but not in the mild group. P = 0.002 for control vs severe, p = 0.52 for control vs mild by Dunnett’s test. B) Chamber area ratio is significantly decreased in severe group, but not in the mild. P = 0.002 for control vs severe, p = 0.25 for control vs mild by Dunnett’s test. N= 15 control, 16 mild, and 7 severe.

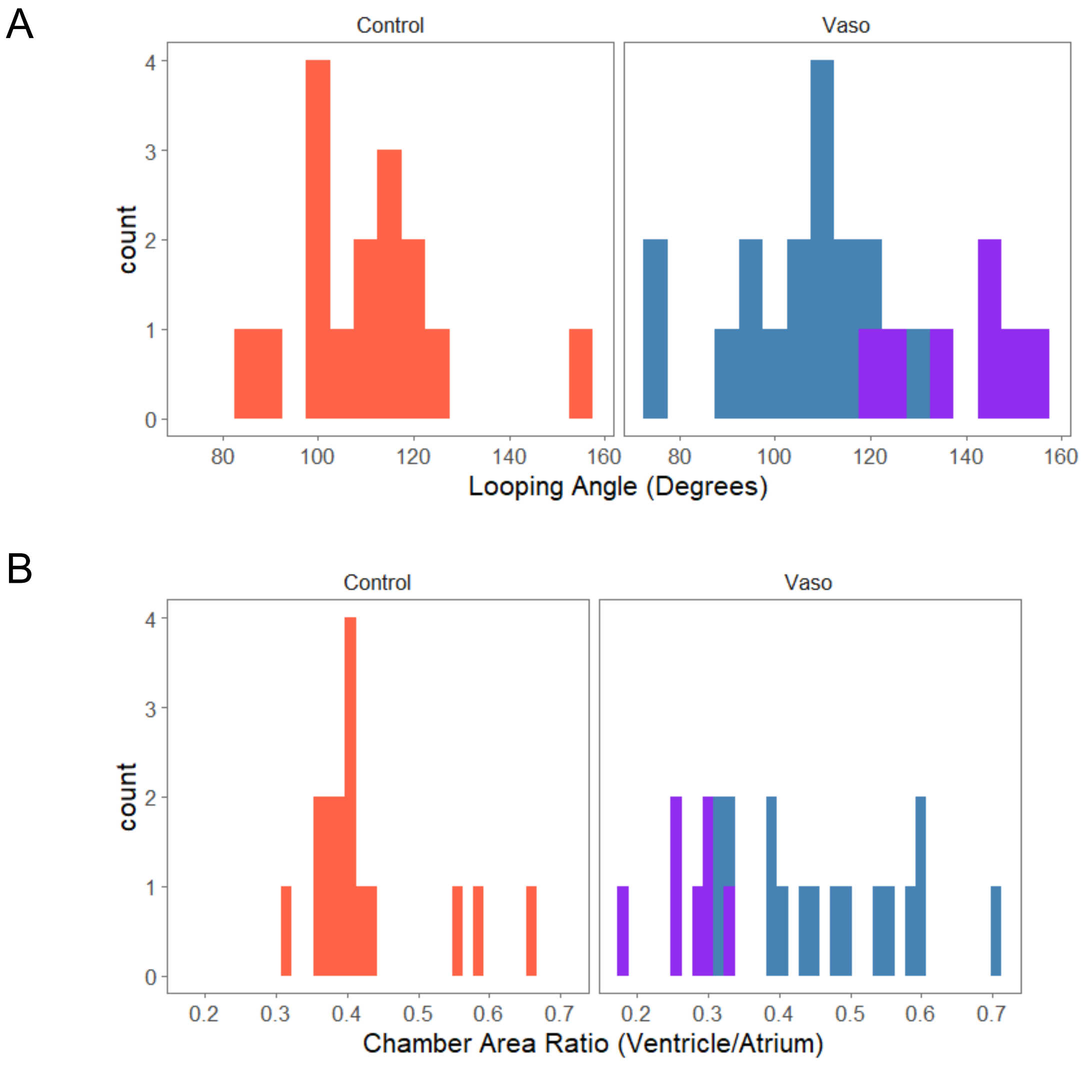

Supplementary Figure S7: In 10 um vasopressin treatments, the severely affected embryos cluster separately from the mildly affected group. A) Histograms of number of embryos vs looping angle or B) chamber area ratio in control and vasopressin-treated groups. Orange represents control embryos, blue represents mildly affected embryos, and purple represents severely affected embryos. N= 15 control, 16 mild, and 7 severe vasopressin-treated embryos.

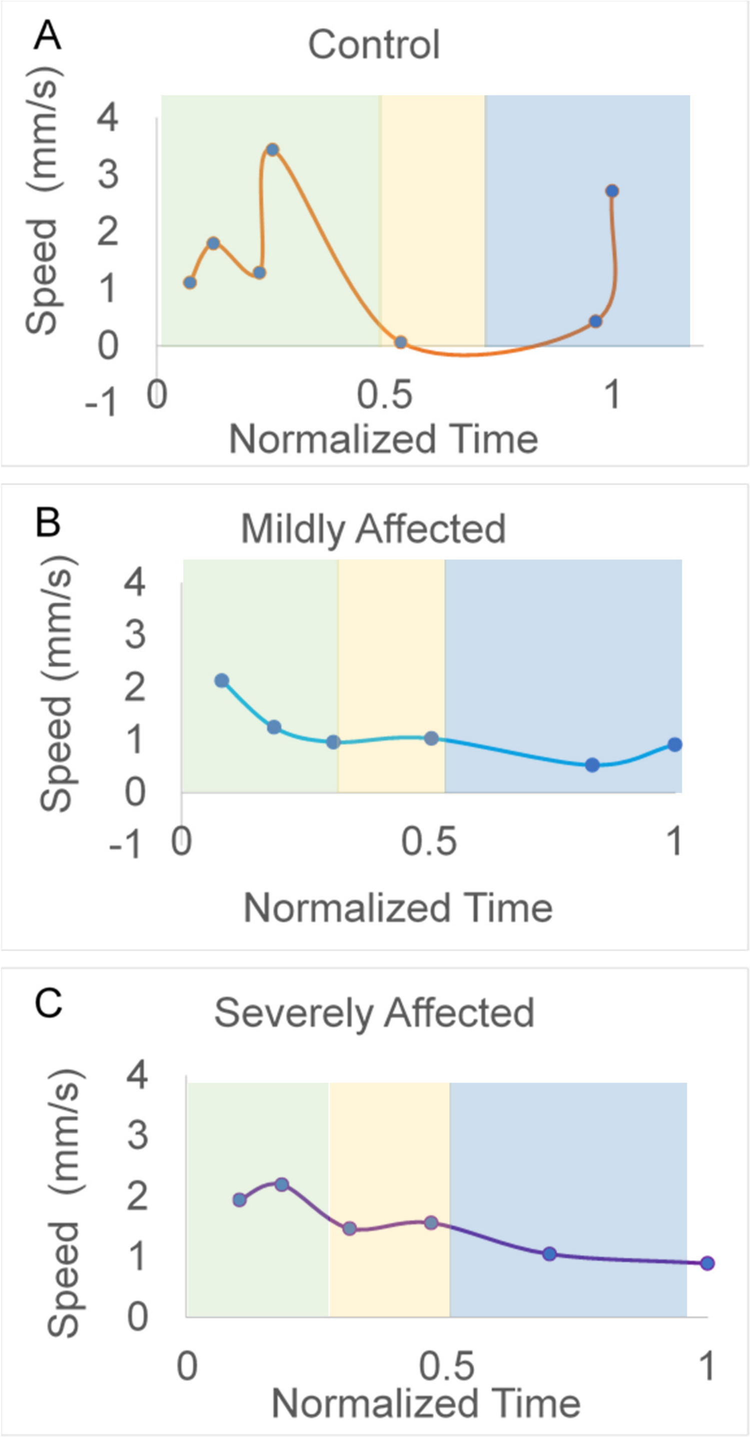

A
Control
Speed (mm/s)
4
3
2
1
0
-1
0
0.5
1
Normalized Time
B
Mildly Affected
Speed (mm/s)
4
3
2
1
0
-1
0
0.5
1
Normalized Time
C
Severely Affected
Speed (mm/s)
4
3
2
1
0
0
0.5
1
Normalized Time

Supplementary Figure S8: Cardiac contraction patterns are altered similarly in mildly and severely affected embryos at 40 hpf. A) Contraction wave of a representative A) control, B) mildly-affected, and C) severely affected 10 μMvasopressin-treated embryo. n = 11 control , 15 mildly-affected, and 5 severely-affected embryos. 0 normalized time represents start of atrial contraction. Green shading represents the atrium, yellow represents the AVJ, and blue represents the ventricle.

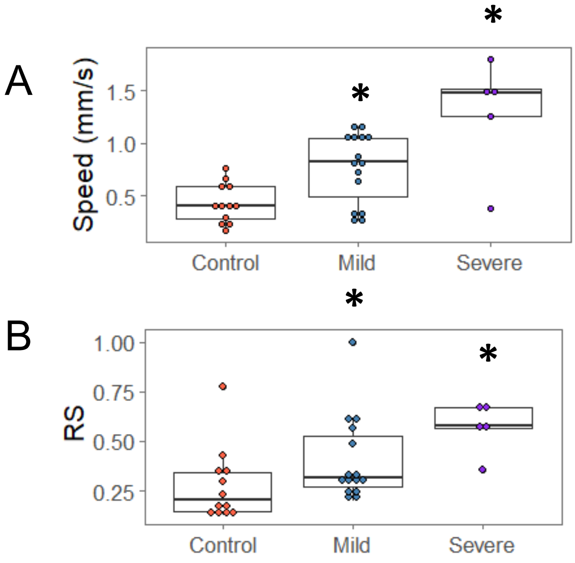

Supplementary Figure S9: The speed of the contraction through the AVJ is significantly increased in both mildly-affected, and severely-affected vasopressin-treated embryos. A) Speed of the contraction wave through the AVJ. B) Relative speed of the contraction wave – results have been normalized to the peak velocity of the contraction wave. N= 11 control, 15 mild, and 7-severe vasopressin-treated embryos. For the speed of contraction wave, p = 0.03 for control vs mild, and p =0.003 for control vs severe by Dunnett's test. For the relative speed, p =0.04 for control vs mild, p =0.003 for control vs severe.

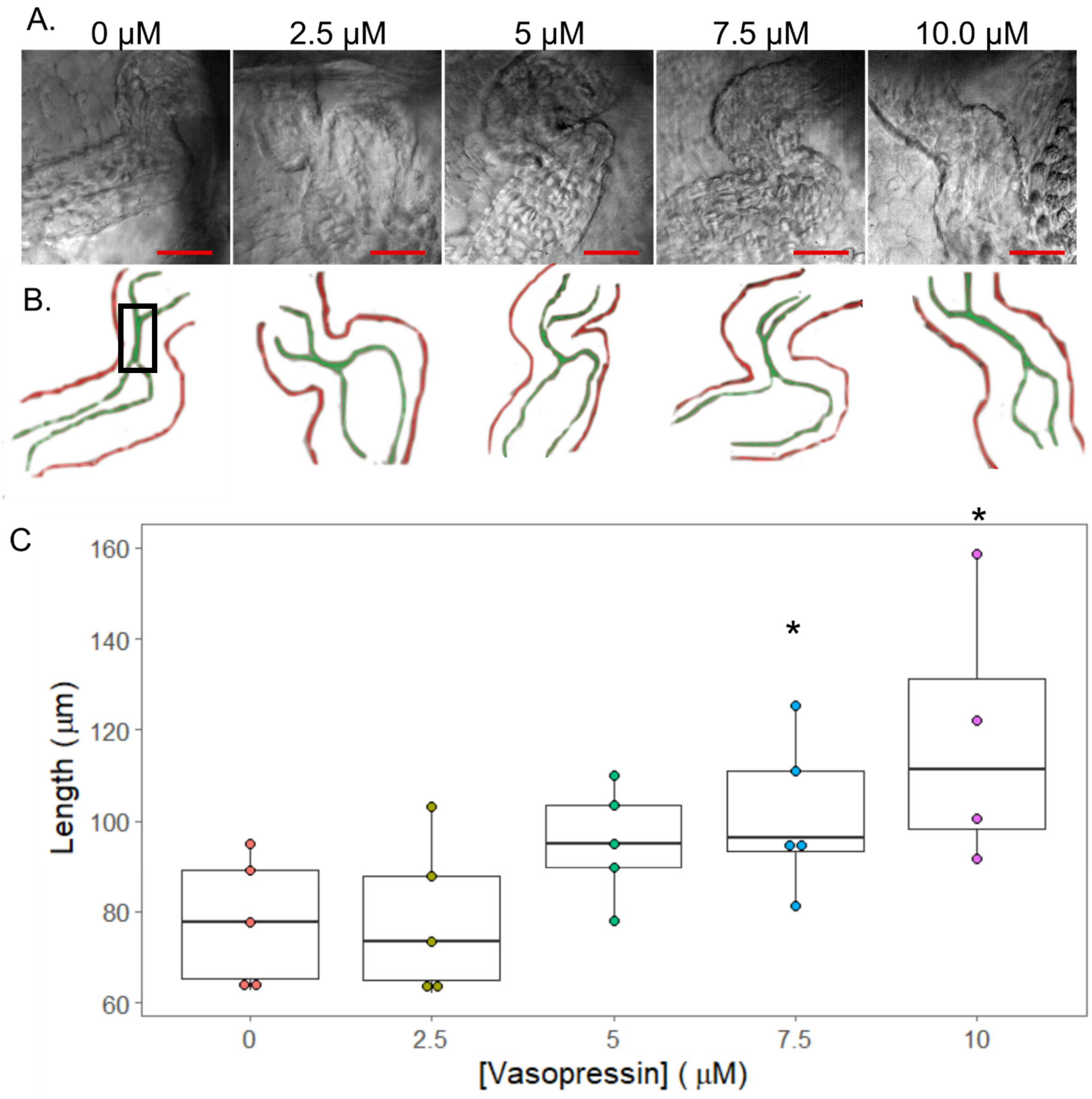

Supplementary Figure S10: Length of endothelial closure increases in a dose-dependent manner in response to afterload at 40 hpf. A) Endothelial closure at the end of ventricular systole at varying doses of vasopressin. B) Tracings of hearts. Red represents outer (myocardial) wall, green represents inner (endocardial) layer. The regions where the endocardial layers meet was quantified as the "length of endocardial closure" in C. Black box in control panel indicates the region being measured. C) Length of endothelial closure increases as vasopressin concentration increases. ANOVA was used to determine statistical significance, followed by Tukey's test. * indicates $p < 0.05$. Control vs 2.5 μM, $p = 0.99$; control vs 5 μM, $p = 0.57$; control vs 7.5 μM, $p = 0.04$, control vs 10 μM, $p = 0.01$.

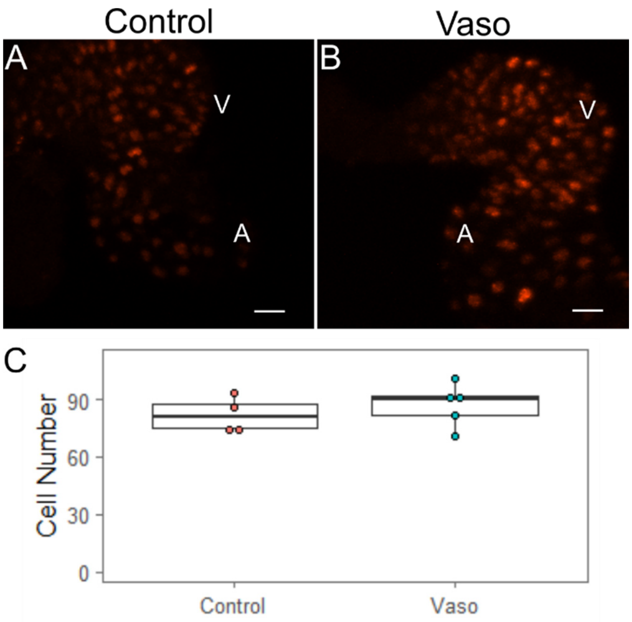

Supplementary Figure S11: Ventricular cardiomyocyte cell number is not affected by vasopressin-treatment. Maximum intensity projections of A) Control and B) vaso-treated embryos at 56 hpf. n = 4 control and 5 vaso-treated embryos. P = 0.48 V denotes ventricle, while A indicates atrium.

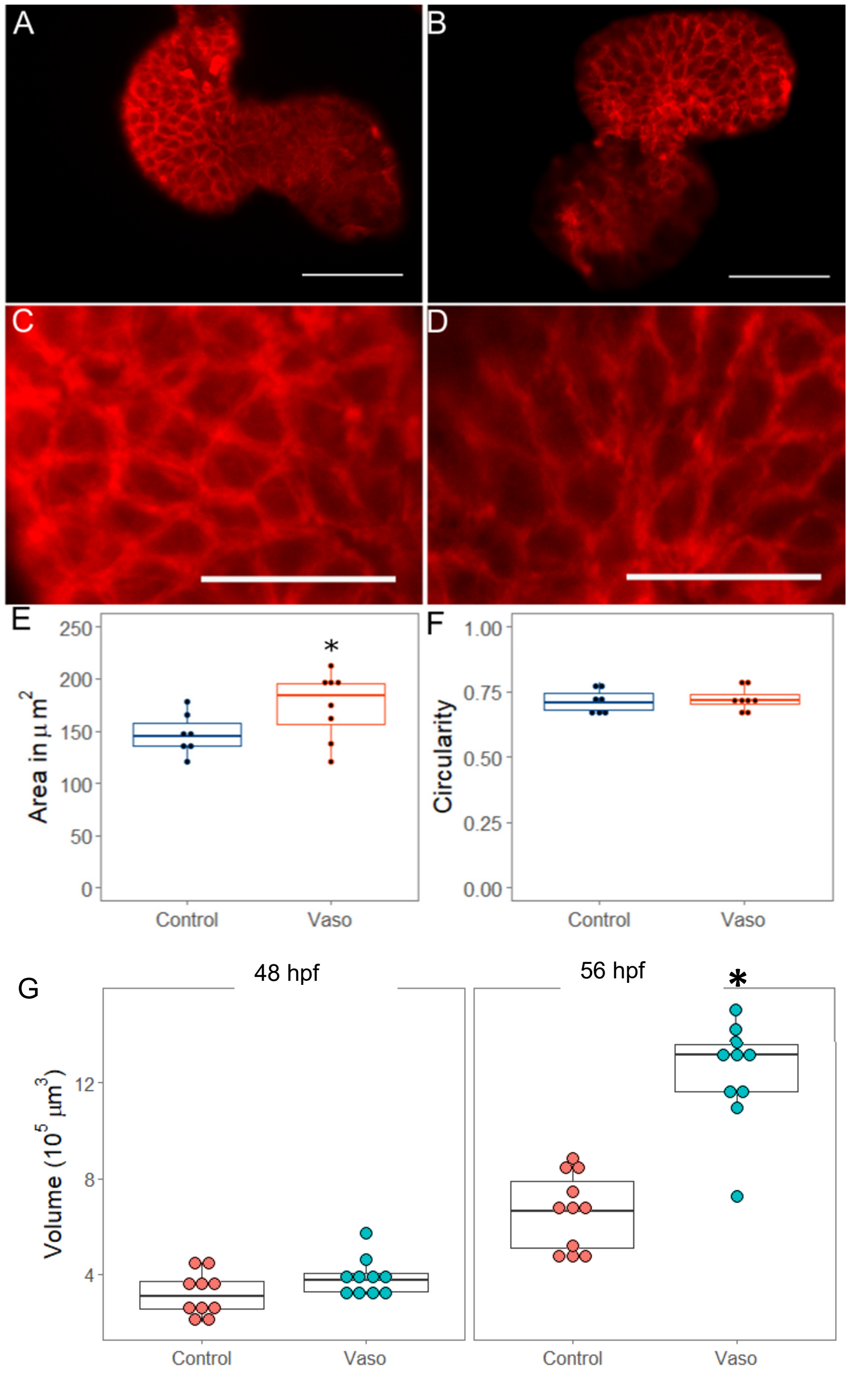
A
B
C
D
E
F
250
200
150
100
50
0
Area in μ m^2
*
Control
Vaso
1.00
0.75
0.50
0.25
0.00
Circularity
G
48 hpf
56 hpf
Volume (10^5 μm^3)
12
8
4
*

Supplementary Figure S12: Application of vasopressin mimics known effects of increased afterload. A) Control heart at 52 hpf. B) Vasopressin-treated heart at 52 hpf. Scale bar represents 20 μm. C) Control ventricle at 52 hpf. D) Vasopressin-treated heart at 52 hpf. Scale bar represents 20 μm. E) Area and F) Circularity of myocardial cells in the ventricle of control and vaso-treated hearts.N= 7 for wildtype hearts, n =7 for vasopressin-treated hearts. 5 myocytes were analyzed per heart. Student's T-test was used to determine statistical significance, * indicates $p < 0.05$. **G.** Chamber all volume is significantly increased in vaso-treated hearts by 56 hpf. n = 10 fish per group (vasopressin treated or control) per time point. A Student's T-test was used to determine statistical significance, * indicates $p < 0.05$.

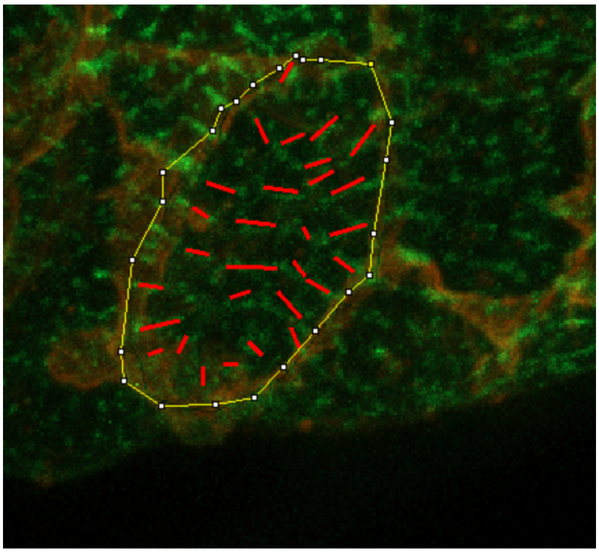

Supplementary Figure S13: Example of how total z-band size /cell (TBZ) was calculated. Red lines trace the GFP-labeled z discs, while the yellow line traces the myocardial cell border. Both the cross-sectional area of the cell and the length of each z- band was quantified in Image J. The individual lengths of z- band were summed together in each cell measured, this was termed "Total Z-band Size." Total z-band size was then divided by the area of the cell to normalize for cell size differences. Over 2000 z-bands were measured in both the control and vasopressin-treated groups.

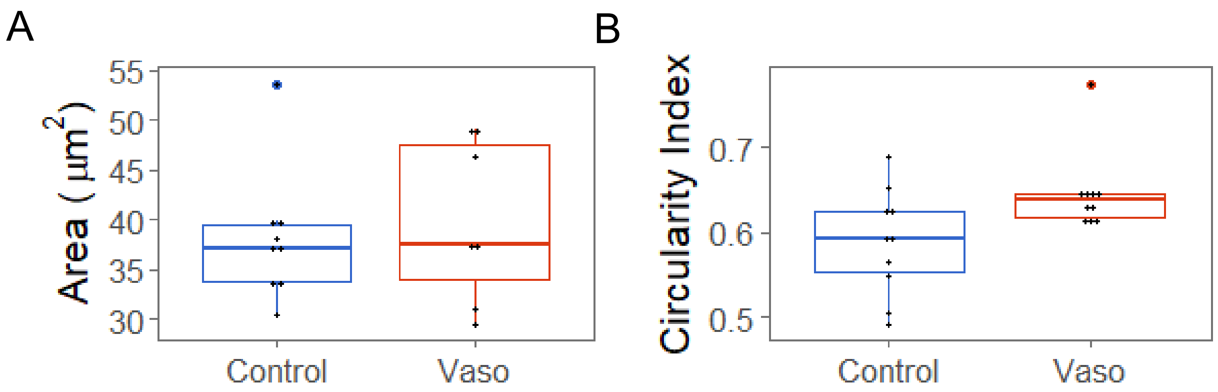

Supplementary Figure S14: Myocardial AVJ cells adopt altered morphology in response to high afterload. N = 11 wildtype hearts, 10 vasopressin treated hearts. Statistical significance was determined using Student's T-test; * indicates $p < 0.05$. A) and B) Analysis of myocardial cells. 5 cells were analyzed per location per heart, area was measured for each cell and averaged. AVJ myocardial cells were defined as myocardial cells located in the constriction between the atrium and ventricle.

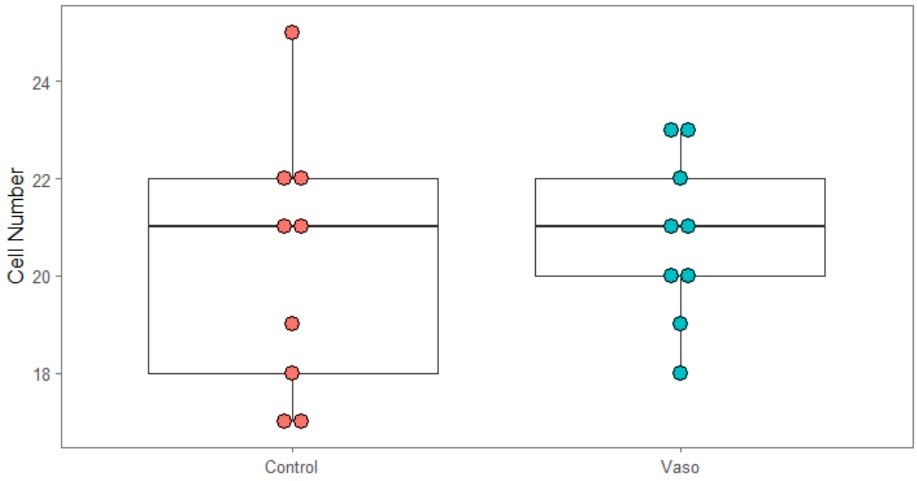

Supplementary Figure S15: Total endocardial cells located in AVJ is not affected by vasopressin-treatment at 72 hpf. AVJ endocardial cells were defined as fli-positive cells located in the constriction between the atrium and ventricle. P=0.60.

Detailed Methods

Zebrafish Husbandry and Strains

Zebrafish were raised as per Colorado State University Animal Care and Use Protocols. Specifically, adult zebrafish are maintained in a fish facility with recirculating water, at an approximate temperature of 28°C. For all non-transgenic experiments, the WIK line was used.[1] For transgenic experiments, Tg(Fli1:eGFP; gata1:dsRED), Tg(myl7:cypher-egfp), Tg(myl7:dsRed-nuc), and Tg(myl17:eGFP) lines were obtained from ZIRC. Embryos were kept in E3 medium supplemented with methylene blue at a density of no more than 60 embryos/20mL E3 in a petri dish.

At 24 hpf, embryos were screened and any embryos that showed gross morphological or developmental defects were discarded. Embryos were then dechorionated and randomly assigned into either the control or treatment groups. Simple randomization was used to assign embryos into either control or treatment groups. Details regarding sex of the embryos used are not provided because, at the stages of interest, the sex of zebrafish embryos has not yet been determined.[2] Therefore, it is not possible to control for this variable this early on in development.

All zebrafish procedures were approved by the Institutional Animal Care and Use Committee at Colorado State University.

Immunohistochemistry

Briefly, hearts were dissected in an L15-10% FBS solution and then placed on a polylysine-coated slide. Hearts were fixed for 40 minutes in 4% PFA and permeabilized with a 1% solution of Triton-X. For ALCAM labelling, zn8 antibody (Hybridoma Bank) was used at 1:10 dilution. Embryos were washed 3X with PBST solution and then incubated with Alexa-546 anti-mouse IgG secondary antibody (Thermo-Fischer Cat #A-11030) at a 1:200 dilution. Hearts were mounted in 50% glycerol under coverslips. Images were taken on the Zeiss LSM800 and analyzed blind.

Quantitative Polymerase Chain Reaction

Hearts were dissected in L15-10% FBS. 10-20 hearts per treatment were pooled, and RNA was extracted using TRIzol (Invitrogen) according to the manufacturer's protocol. cDNA synthesis was performed using AMV reverse transcriptase (Fisher Scientific) and Oligo(dT)$_{12–18}$ primer (Invitrogen). Resulting cDNA was treated with RNase H (New England Biolabs, Ipswich, MA). Standards curves were performed in triplicate for every gene. Reactions were run on Roche LC480 qPCR machine. Accompanying software (Roche LightCycler 480 software, version 1.2) was used to calculate Ct values. Pfaffl analysis was used to determine relative expression.[3] In all experiments, the reference gene used was *Elongation Factor α (EF α)*, a well validated housekeeping gene.[4]

Live Fluorescence Imaging

For vessel, samples were immobilized in agarose and treated with MESAB to temporarily stop heartbeats. Samples were visualized using an Olympus SZX12 Fluorescent stereo-dissecting microscope. Images were taken using a QI-imaging camera Quantification of vessel diameter was performed in Image J.[5] Quantification of cardiac chamber volume and looping angle was performed as previously described.[6,7]

Quantification of Cardiac Chamber Volume and Looping Angle

After being randomly assigned to either control or treatment groups, embryos were incubated until 56 hours post fertilization. Embryos were then treated with tricaine, and incubated until their hearts stopped. They were then mounted in low-melt agarose such that both chambers of their heart were in focus, ventral-side up. They were then imaged immediately under an Olympus SZX-12 stereomicroscope. Image analysis was performed in ImageJ.

To measure cardiac chamber volume, the both the length and width of the chamber were measured. Volume of the chamber volume was determined using the following formula: $V = \pi r^2 h$, where r was defined as the width of the ventricle and h was defined as the length. To measure looping angle, the angle between the line parallel to blood flow through the AVJ and bisecting the atrium was quantified in imageJ.[8]

Quantification of Total Z-band Size

To visualize cell membranes, Tg(*myl7*:cypher-egfp) hearts underwent immunohistochemistry as described in the methods section. Z-stacks spanning the entire heart were taken on the Zeiss LSM 800 confocal microscope and processed in Image J Fiji. Briefly, 5 cells from the region of interest (either the outer curvature or AVJ) were selected and traced using the polygon tool to measure area. Each of these cells was then analyzed for total Z-band size. In each slice of the z-stack, the z-bands were traced and added to the ROI manager. The entire cell through 3-dimensions was scanned for z-bands, all z-bands were traced. The length of each z-band was measured and summed to determine total z-band size.

Quantification of the Contractile Wave

To quantify the contractile wave in each heart, videos were taken as described in the methods section. Image sequences were opened in Image J Fiji. First, total frames per cycle was determined by finding the number of frames between successive atrial inlet contractions. The point where the lumen was closest together was manually tracked at throughout the cardiac cycle, and distance between the two points was measured by manually tracing the path of contraction and noting the distance and frame number between each point. Frame number was converted to time, and speed of the contractile was quantified as distance over time. Velocity was plotted over normalized time, defined as ((current frame number – starting frame number)/ total frames per cycle)).

Quantification of Cardiomyocyte Cell Number

To quantify cardiomyocyte cell number, hearts from Tg(myl7:dsred-nuc) embryos were dissected as previously described.[9] Hearts were then exposed to 4% PFA for 40 minutes, and washed 3 times with PBT. Hearts were mounted in 50% glycerol and imaged on the LSM800. Images were made into maximum intensity projections using ImageJ and ventricular cells were manually counted.

In Situ Hybridization

In situ hybridization was carried out as previously described.[10] In brief, zebrafish embryos at the appropriate stage were fixed in 4% paraformaldehyde at 4°C overnight. Embryos were then placed in 100% methanol, and placed in -20°C for at least 2 hours. Embryos were then gradually rehydrated from methanol to phosphate buffered saline with 1% Tween-20. Embryos

were then permeabilized with proteinase K for 20 minutes at room temperature. Embryos were then placed in 4% paraformaldehyde for 20 minutes at room temperature, and washed 5 times with phosphate buffered saline. Embryos were then incubated with DIG-labeled probe of interest in hybridization mixture (50% formamide, 5x saline sodium-citrate solution, 1% Tween-20) overnight at 60°C. Embryos were then washed with sodium citrate solution and PBT to remove all unbound probe. Embryos were then incubated with secondary antibody (Anti-Digoxigenin-AP Fab fragmants, Roche) at a concentration of 1:5000 overnight in 4°C. Embryos were then washed with PBT several times to remove unbound secondary antibody. Embryos were then placed in a staining solution consisting of 1% 4-Nitro blue tetrazolium chloride (Roche) and 0.7% 5-Bromo-4-chloro-3-indoyl-phosphate (Roche). Embryos were kept in the dark and monitored for signal development. After the controls developed adequate signal, reactions were stopped by placing embryos in 4% paraformaldehyde.

Detailed Statistical Analysis

All data was analyzed in R (version 3.4.2), and graphs were made using the ggplot2 package. Data was first checked for normality using shapiro-wilk function built into base R. If the shapiro-wilk test returned a p-value greater than 0.05, then a two tailed t-test was used to analyze the data. If the shapiro-wilk test returned a value less than 0.05 and the number of replicates were less than 40, than the Kruskal-wallis test was used to determine statistical significance. For multiple comparisons, Tukey''s test was used for parametric data and Dunnet's many-against-one test was used for nonparametric data.

References

1. Rauch G-J, Granato M, Haffter P. A polymorphic zebrafish line for genetic mapping using sslps on high-percentage agarose gels. 1997
2. Liew WC, Orbán L. Zebrafish sex: A complicated affair. *Brief Funct Genomics*. 2014;13:172-187
3. Pfaffl MW. A new mathematical model for relative quantification in real-time rt–pcr. *Nucleic Acids Research*. 2001;29:e45.
4. McCurley AT, Callard GV. Characterization of housekeeping genes in zebrafish: Male-female differences and effects of tissue type, developmental stage and chemical treatment. *BMC Mol Biol*. 2008;9:102
5. Rueden CT, Schindelin J, Hiner MC, DeZonia BE, Walter AE, Arena ET, Eliceiri KW. Imagej2: Imagej for the next generation of scientific image data. *BMC Bioinformatics*. 2017;18:529
6. Yang J, Hartjes K, Nelson T, Xu X. Cessation of contraction induces cardiomyocyte remodeling during zebrafish cardiogenesis. *Am J Physiol Heart Circ Physiol*. 2014;306:382-395
7. Berndt C, Poschmann G, Stühler K, Holmgren A, Bräutigam L. Zebrafish heart development is regulated via glutaredoxin 2 dependent migration and survival of neural crest cells. *Redox Biol*. 2014;2:673-678
8. Johnson B, Garrity D, Dasi P. The transitional cardiac pumping mechanics in the embryonic heart. *Cardiovascular Engineering and Technology*. 2013;4:246-255
9. Yang J, Xu X. Immunostaining of dissected zebrafish embryonic heart. *J. Vis. Exp.* 2012

10. Thisse C, Thisse B. High-resolution in situ hybridization to whole-mount zebrafish embryos. *Nat Protoc*. 2008;3:59-69
